# Current biogeographical roles of the Kunlun Mountains

**DOI:** 10.1002/ece3.8493

**Published:** 2022-01-15

**Authors:** Weibo Du, Peng Jia, Guozhen Du

**Affiliations:** ^1^ School of Life Sciences State Key Laboratory of Grassland and Agro‐Ecosystems Lanzhou University Lanzhou China

**Keywords:** biogeographical roles, evolutionary history, flora, geological evolution, Kunlun Mountains, phylogenetic structure, phylogeography, plant community, plant diversity, seed plants

## Abstract

Large‐scale patterns of biodiversity and formation have garnered increasing attention in biogeography and macroecology. The Qinghai‐Tibet Plateau (QTP) is an ideal area for exploring these issues. However, the QTP consists of multiple geographic subunits, which are understudied. The Kunlun Mountains is a geographical subunit situated in the northern edge of the QTP, in northwest China. The diversity pattern, community phylogenetic structures, and biogeographical roles of the current flora of the Kunlun Mountains were analyzed by collecting and integrating plant distribution, regional geological evolution, and phylogeography. A total of 1911 species, 397 genera, and 75 families present on the Kunlun Mountains, of which 29.8% of the seed plants were endemic to China. The mean divergence time (MDT) of the Kunlun Mountains flora was in the early Miocene (19.40 Ma). Analysis of plant diversity and MDT indicated that the eastern regions of the Kunlun Mountains were the center of species richness, endemic taxa, and ancient taxa. Geographical origins analysis showed that the Kunlun Mountains flora was diverse and that numerous clades were from East Asia and Tethyan. Analysis of geographical origins and geological history together highlighted that the extant biodiversity on the Kunlun Mountains appeared through species recolonization after climatic fluctuations and glaciations during the Quaternary. The nearest taxon index speculated that habitat filtering was the most important driving force for biodiversity patterns. These results suggest that the biogeographical roles of the Kunlun Mountains are corridor and sink, and the corresponding key processes are species extinction and immigration. The Kunlun Mountains also form a barrier, representing a boundary among multiple floras, and convert the Qinghai‐Tibet Plateau into a relatively closed geographical unit.

## Graphical abstract

The biogeographical roles of the Kunlun Mountains were corridor and sink, and the corresponding key processes were species immigration and extinction. The extant biodiversity on the Kunlun Mountains has occurred through species recolonization after climatic fluctuations and glaciations during the Quaternary. The Kunlun Mountains also formed a barrier, representing a boundary among multiple floras, and converted the QTP into a closed physical geographical unit. The nearest taxon index indicated that habitat filtering may have played an important role in biodiversity patterns.

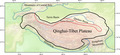

## 1 INTRODUCTION

Studying biodiversity patterns at the regional scale has always been a research focus in macroecology and biogeography (Grierson et al., 2011; Patino et al., 2017). Numerous studies have suggested that contemporary environmental factors (e.g., climate and habitat heterogeneity) and historical processes (e.g., speciation, extinction, and dispersal) jointly influence biodiversity (Brown et al., 2004; Currie et al., 2004; Kerr & Packer, 1997; Mittelbach et al., 2007; Ricklefs, 2005; Wang, Brown, et al., 2009; Zobel, 1997). However, to date, no theory has integrated the relative effect of contemporary environments and historical processes on biodiversity patterns (Hawkins & Porter, 2003; Montoya et al., 2007; Svenning & Skov, 2005; Wang et al., 2012). In addition, the potential linear correlation between contemporary environments and historical processes is difficult to distinguish from their respective value. Therefore, it is particularly important to independently explore contemporary environments and historical processes.

Integration analysis of taxonomy, phylogeny, ecology, biogeography, phylogeography, and paleontology may offer an insightful perspective on the biodiversity patterns at different scales (Li, Qian, Sun, 2018). Ecologists and biologists have analyzed multiple regions to independently explore historical processes of biodiversity patterns. For example, the formation time of the Andes flora was 6.40 Ma (Chen et al., 2018; Särkinen et al., 2012); they present biogeographical roles are cradles of alpine flora, a dispersal barrier to lowland species, and a dispersal corridor for South and North American species (Antonellia et al., 2009; Luebert & Weigend, 2014). The Andean uplift has been vital for the evolution of the Amazonian flora, with a formation time of 8.30 Ma (Chen et al., 2018; Hoorn et al., 2010). The formation time of the Australian flora was estimated to be 18.80 Ma (Chen et al., 2018; Crisp & Cook, 2013). Their biogeographical roles are reservoirs of ancient taxa and sinks of recent lineages (Crisp & Cook, 2013). The formation time of the South African flora was 18.70 Ma (Chen et al., 2018; Linder & Verboom, 2015). Speciation and dispersal played a leading role in biodiversity in the area (Linder & Verboom, 2015). According to the analysis on the angiosperm flora of China, the eastern China act as museums and cradles of woody species, and the western China act as cradles of herbaceous species (Lu et al., 2018).

Studies have widely recognized that the abiotic environment, contemporary biotic interactions, and evolutionary history jointly explain community assembly at different scales (Cavender‐Bares et al., 2009; Ricklefs, 2006; Vellend, 2010). The phylogenetic community structure can be used to explore ecological and evolutionary processes of community assembly at different scales (Kraft et al., 2007; Webb et al., 2002). Evolutionary processes such as rapid *in situ* speciation, niche conservatism, and dispersal limitation can lead to phylogenetic clustering (Lu et al., 2018). In comparison, evolutionary processes such as niche evolution, convergent evolution, and colonization may lead to phylogenetic overdispersion within communities (Allen & Gillooly, 2006). Ecological processes, habitat filtering, and competitive exclusion can result in nonrandom community phylogenetic structures (Kraft et al., 2007; Webb et al., 2002). Habitat filtering can lead to phylogenetic clustering, the process select species with similar functional traits into a community (Wiens & Graham, 2005), whereas a community dominated by competitive exclusion might show phylogenetic dispersion (Burns & Strauss, 2011).

With topographically complex mountains, the biodiversity and ecosystem processes between mountains and adjacent lowlands are influenced by biotic interchange, regional climate, and nutrient runoff (Rahbek, Borregaard, Antonelli, et al., 2019). In addition, mountains reportedly disproportionately influence the global terrestrial biodiversity, especially in the tropics, where they harbor extraordinarily rich species. Generally, the mountains of the arctic and temperate regions have few endemic species and low species diversity; biodiversity of these mountains barely exceeds that of the adjacent lowlands (Rahbek, Borregaard, Colwell, et al., 2019). In addition, at a large spatial and temporal scale, geological history and abiotic environment jointly regulate four key processes that determine the biodiversity worldwide: speciation, dispersal, persistence, and extinction (Rahbek, Borregaard, Antonelli, et al., 2019). Consequently, mountains are ideal regions for exploring the mechanisms that govern biodiversity patterns at different scales. Based on the different processes, mountains are classified as having different biogeographical roles (Rahbek, Borregaard, Antonelli, et al., 2019).

The high mountains of China are mainly located in the Qinghai‐Tibet Plateau (QTP) and adjacent regions (Wang et al., 2004). The QTP is the plateau itself, which is the largest and highest plateau in the world, occupying an area of 2.5 million km^2^, with an average elevation of over 4000 m (Zhang et al., 2002). The datasets thereby accumulated from studies that have been conducted on the QTP offer opportunities to investigate the biodiversity patterns and plant communities in the regions (Favre et al., 2015). According to data in published monographs and literature, the QTP possesses ~10,000 species of vascular plants (APGIV, 2016; Wu, 2008), of which ~20% are endemic to the region (Wu, 2008; Yan et al., 2013; Yu, Zhang, et al., 2018). Further, species richness varies considerably across the region (Mao et al., 2013; Yan et al., 2013), with the southern regions having especially high species richness (Mao et al., 2013). Rapid speciation and habitat filtering have been reported to dominate the biodiversity and community assembly processes on the QTP, and the phylogenetic structure of vascular species is clustered in most regions of the QTP (Yan et al., 2013). The geological history and uplifts of the QTP are still being debated because the QTP consists of multiple geographical subunits that have experienced different geological events and uplifts (Deng et al., 2017; Renner, 2016; Spicer et al., 2020; Su et al., 2019; Sun & Zheng, 1998). However, previous studies indicate that the QTP has risen to its current elevation only in the late Neogene (23.3 Ma–2.6 Ma) (Li et al., 2021; Spicer et al., 2020; Su et al., 2019). There is a consensus that the QTP has undergone strong climatic fluctuations and four major glacial events during the Quaternary (Owen & Dortch, 2014; Renner, 2016; Shi et al., 1998). These geological processes at the QTP have promoted radiation and species diversification in various plants taxa (Wen et al., 2014), and caused mass plant extinction.

Owing to major advancements in phylogeographic studies and tools, the numerous plant speciation and adaptations in the QTP and adjacent regions, such as *Saussurea* (Wang, Susanna, et al., 2009), *Rheum* (Sun et al., 2012), *Gentiana* (Favre et al., 2016), *Rhodiola* (Zhang et al., 2014), *Saxifraga* (Ebersbach et al., 2017), and *Syncalathium* (Zhang et al., 2011), among others (Liu et al., 2017; Qiu et al., 2011), have been increasingly reported (Liu et al., 2014). These datasets provide the opportunity to explore the biodiversity formation and maintenance mechanisms in these areas. Datasets from different subunits have driven further exploration of the plant diversity on the QTP. For example, the Hengduan Mountains have acted as cradles, refugia, and independent biogeographic sources since the Neogene (Ding et al., 2020; Liu et al., 2017; Muellner‐Riehl, 2019; Sun et al., 2017; Xing & Ree, 2017). The alpine flora of the Hengduan Mountains is the largest source of species dispersal for the Himalayas and the QTP (Ding et al., 2020). Recent studies have shown that the main phylogeographic patterns of seed plants include contraction/recolonization, platform refugia/local expansion, and microrefugia in the Tibeto‐Himalayan region (Muellner‐Riehl, 2019).

Most previous researches have focused on the QTP as a whole, and there has been little research on the geographical subunits in the region. The Kunlun Mountains are a geographical subunit with a relatively clear geographical range and available plant distribution data; however, they are not considered as a biodiversity hotspot and seem to harbor few species (Pan, 2000; Su, 1998; Sun et al., 2015; Wu, 2012–2015; Zachos & Habel, 2011; Zheng, 1999;). With respect to the phytogeographical regions of the Chinese flora, the Kunlun Mountains form the border between the Tethyan region and the QTP (Ye et al., 2019, 2020). In addition, they present the richest contemporary glaciers in China (Liu et al., 2015). Therefore, we used datasets to explore the biogeographical roles of the Kunlun Mountains to: (1) clarify patterns of the extant taxa diversity on the Kunlun Mountains; (2) estimate the formation time and the geographical origin of the Kunlun Mountains flora; and (3) explore the driving forces underlying the formation of the Kunlun Mountains flora. Our findings could further aid reveal biogeographical roles.

## 2 MATERIALS AND METHODS

### 2.1 Study regions

**FIGURE 1:**
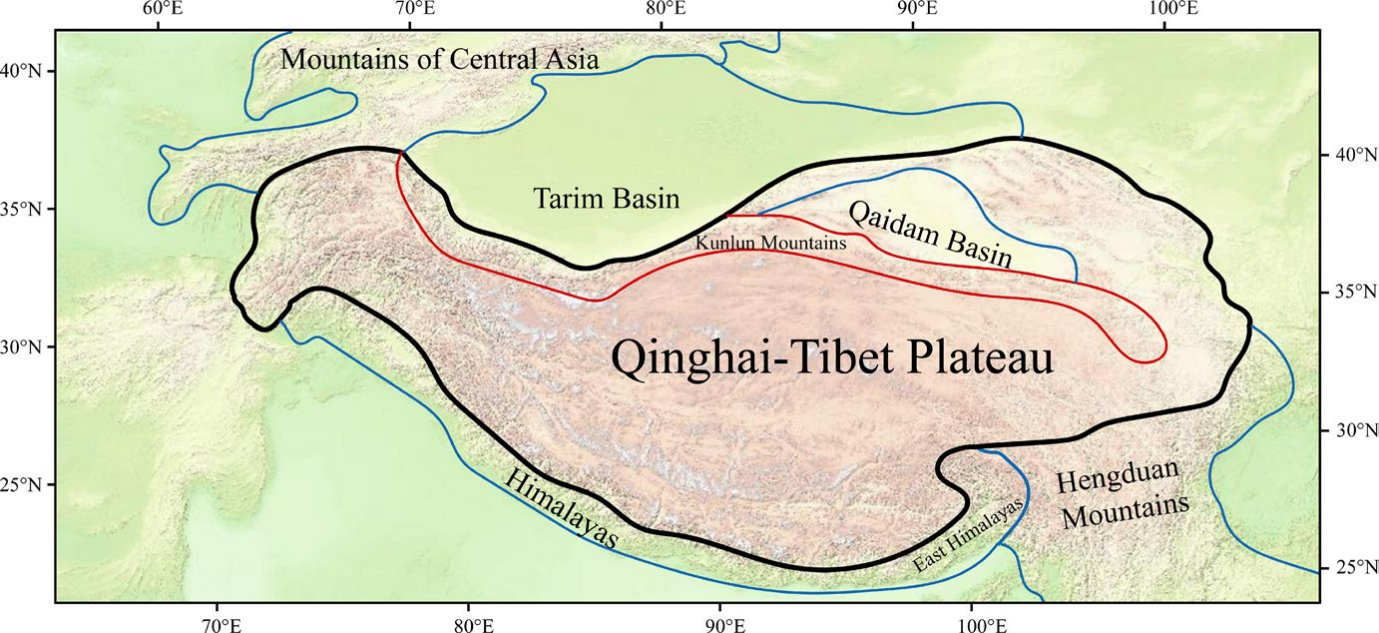
Position relations of the Kunlun Mountains and around geographic units

The Kunlun Mountains are a geographical subunit located in northwest China, on the northern edge of the QTP. Their range lies across 34°N–40°N and 75°E–100°E. Geographically, they extend to southeast Qinghai in the east, the Pamirs Plateau in the west, the northwest Tibet Autonomous Region in the south, the Qaidam and Tarim basins in the north. The line of the mountains is east–west, and broader in the east than in the west. The Kunlun Mountains are a total length of ~2500 km and a width of 130–200 km, an area of over 500,000 km^2^ (Wu, 2012–2015; Figure 1). Their elevation gradually rises from the east to the west, and ranges between 3000 m and 7719 m, with an average altitude of approximately 4000 m.

The annual precipitation and average annual temperature of the regions vary from ~100 to 500 mm and below 0°C, respectively. The annual precipitation is characterized by a decrease from the east to the west. The climate varies also on the slopes of the mountains, with a steep climate gradient leading to a dramatic change in vegetation cover. The dominating vegetation types are alpine meadow and alpine steppe, a few alpine scrubs and coniferous forests distribute in the east and west of the Kunlun Mountains (Wu, 2012–2015; Zheng, 1999).

The uplift of the Kunlun Mountains coincided with the Himalayan movement, and its geological history and uplifts are still unknown (Duvall et al., 2013; Jiang et al., 2013; Wang et al., 2003; Wang & Chang, 2012; Yin et al., 2008). However, it is certain that the climatic fluctuations and glaciations of the Quaternary also occurred in the Kunlun Mountains (Owen et al., 2008; Owen & Dortch, 2014; Renner, 2016).

**FIGURE 2:**
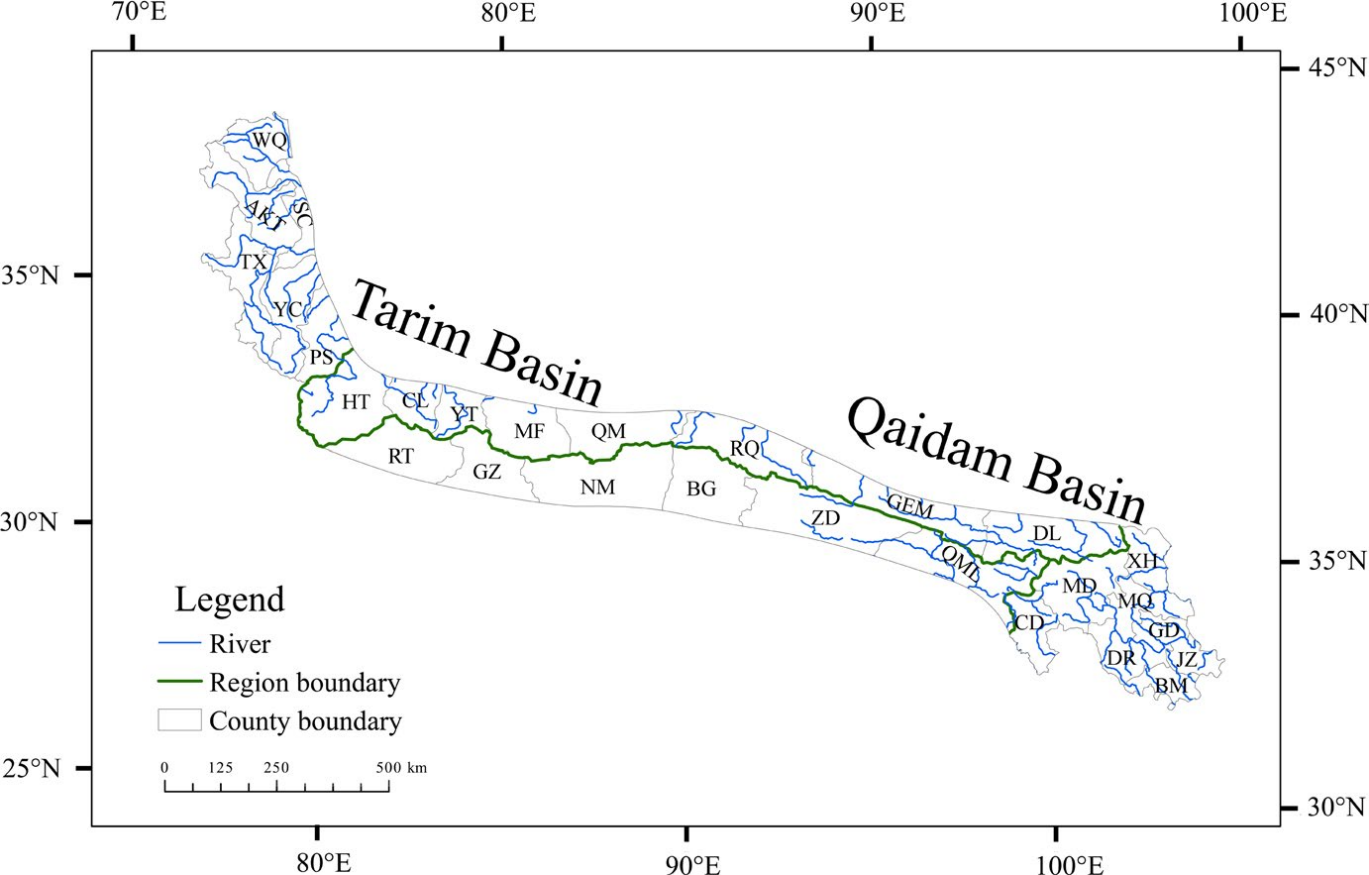
The division of county‐level and regional units in the Kunlun Mountains, China

To accurately analyze the regions, the Kunlun Mountains was classified into 28 county‐level geographical units dependent on vegetation types and the county area. In addition, the Kunlun Mountains are also divided into four regions: east, west, and the southern and northern slopes of the central region. The western region and the southern slope of the central region each consist of six counties, and the northern slope and the eastern region each consist of eight counties (Figure 2; Table 1).

**TABLE 1:** The plant diversity of 28 counties on the Kunlun Mountains

Counties	Genera/Endemic genera	Endemic species	Species	Counties	Genera/Endemic genera	Endemic species	Species
East Kunlun Mountains				North slope of Central Kunlun Mountains			
Banma (BM)	182/2	167	391	Dulan (DL)	108/0	59	243
Jiuzhi (JZ)	193/5	214	536	Geermu (GRM)	122/2	60	281
Dari (DR)	119/3	107	288	Ruoqiang (RQ)	124/1	45	294
Gande (GD)	73/1	55	153	Qiemo (QM)	81/0	20	157
Chenduo (CD)	179/4	176	490	Minfeng (MF)	51/0	6	69
Maduo (MD)	140/3	145	471	Yutian (YT)	72/0	13	120
Maqin (MQ)	234/5	271	749	Cele (CL)	109/0	15	198
Xinghai (XH)	236/4	240	731	Hetian (HT)	93/0	15	177
Total	337/7	493	1,299	Total	198/2	129	639
South slope of Central Kunlun Mountains				West Kunlun Mountains			
Qumalai (QML)	143/3	111	360	Pishan (PS)	98/0	10	173
Zhiduo (ZD)	76/2	50	162	Yecheng (YC)	160/0	20	388
Bange (BG)	70/1	23	129	Shache (SC)	79/0	5	115
Nima (NM)	50/0	21	109	Taxian (TX)	177/0	27	492
Gaize (GZ)	68/0	14	114	Aketao (AKT)	151/0	12	340
Ritu (RT)	127/0	22	263	Wuqia (WQ)	160/0	11	318
Total	192/3	155	602	Total	245/0	51	813

### 2.2 Species distribution

The species distribution was derived from *Flora Kunlunica* (four volumes) by Wu (2012–2015), with references to relevant local floras, specimens’ information, and other literature, including Tibet Autonomous Region (Wu, 1983–1987), Xinjiang (Shen, 1993–2011), Qinghai (Liu, 1996–1999), the Qinghai‐Tibet Plateau (Wu, 2008), and the National Specimen Information Infrastructure (http://nsii.org.cn/2017/home.php). The genera and families were defined by the Angiosperm Phylogeny Group Ⅳ and relevant monographs of Chinese Vascular Plants (APGIV, 2016; Li, Chen, et al., 2018). All the species names were standardized following the Catalogue of Life Checklist (http://www.catalogueoflife.org/annual‐checklist/2019/) and The Plant List (http://www.theplantlist.org). When species names differed between these databases, we followed The Plant List. The information formed a comprehensive checklist, which only recorded wild seed plants, and preserved infraspecific taxa. To reveal spatial patterns, a species checklist of each county‐level geographical unit was also created using species distribution data.

### 2.3 Geographical origin and divergence time of floras

To reveal the geographical origin and divergence time of the flora, we collected data from published phylogeography of clades, following two principles of data collection: taxa from the Kunlun Mountains flora had to include geographical origin or divergence time of these clades, with the divergence time of these clades being crown age (Table S1). Based on the corresponding data, mean divergence times (MDTs) were calculated as:

$$\text{MDT}=\frac{\left({\text{AGE}}_{1}\times S_{1}\right)+\left({\text{AGE}}_{2}\times S_{2}\right)+\left({\text{AGE}}_{3}\times S_{3}\right)+\ldots +\left({\text{AGE}}_{n}\times S_{n}\right)}{S_{1}+S_{2}+S_{3}+\ldots +S_{n}}$$

where AGE*_i_* is the age of the genus *i* (*i* = 1, …, *n*) in a sample, and *S_i_* is the species number of the genus i in the sample. The MDTs of these clades may be used to explore spatial divergence patterns in a region (Lu et al., 2018). The unit of MDT is Ma, which stands for million years.

The standardized effect size of the mean divergence time (SES‐MDT) of the genera in the sample was calculated as:

$$\text{SES}‐\text{MDT}=\frac{{\text{MDT}}_{\text{observed}}‐{\text{MDT}}_{\text{random}}}{\text{SD}\left({\text{MDT}}_{\text{random}}\right)}$$

where MDT_observed_ represents observed MDT, MDT_random_ represents the expected MDT of the randomized assemblages (*n* = 999), and SD (MDT_random_) is the standard deviation of the MDT for the randomized assemblages. For the youngest quartile, samples with values of SES‐MDT below −1.96 were confirmed as significantly young floras, and for the oldest quartile, samples with SES–MDT values above 1.96 were confirmed as significantly ancient floras (Lu et al., 2018).

We calculated the MDT of clades in county‐level geographical units and four regions and used the proportion of different geographical origins to reveal the divergence time of the Kunlun Mountains flora. To accurately test the representativeness of the data according to the vegetation types, the genera were classified into four types, namely, dominant genera, common genera, occasional genera, and endemic genera. The first two of these represent the vegetation types in the areas. We calculated the proportion of dominant genera and common genera in collected clades.

### 2.4 Phylogenetic structure

The nearest taxon index (NTI) was calculated to reveal the community phylogenetic structure, and to explore possible ecological and evolutionary processes of community assembly (Webb et al., 2002). The NTI was based on the mean nearest taxon distance (MNTD), which show the total of the mean phylogenetic relatedness between each taxon and its nearest relative in a sample. The NTI indicates the structure in the shallower parts of a sample (Webb et al., 2002). The positive NTI values indicate that the community phylogenetic structure is phylogenetically clustered, whereas negative values indicate that the community phylogenetic structure is phylogenetically dispersed. The NTI values were calculated as follows:

$$\text{NTI}=‐1\times \frac{{\text{MNTD}}_{\text{observed}}‐{\text{MNTD}}_{\text{random}}}{\text{SD}\left({\text{MNTD}}_{\text{random}}\right)}$$

MNTD_observed_ is the observations of MNTD, MNTD_random_ represents the mean values of expectations of MNTD in random combination (*n* = 999), and SD (MNTD_random_) is the standard deviations of the MNTD_random_ values in the random combination. The establishment of a null model in MNTD depended on a random selection of the observed number of taxa in each sample 999 times, with all the taxa in the phylogeny acting as the sampling pool.

Phylogenetic analyses require a phylogenetic tree of seed plants, and the phylogenetic tree used in our study was constructed using Phylomatic (http://phylodiversity.net/phylomatic/) with the stored tree data from Zanne et al. (2014). Phylomatic standardizes the species names according to The Plant List (Qian & Jin, 2016). The phylogenetic tree was obtained using the Phylomatic dependent on the Angiosperm Phylogeny Group Ⅳ and standardized species names. Ecological index was calculated by picante packages in R version 3.3.3 (Kembel et al., 2010; R Core Team, 2017).

## 3 RESULTS

### 3.1 Patterns of species diversity

**FIGURE 3:**
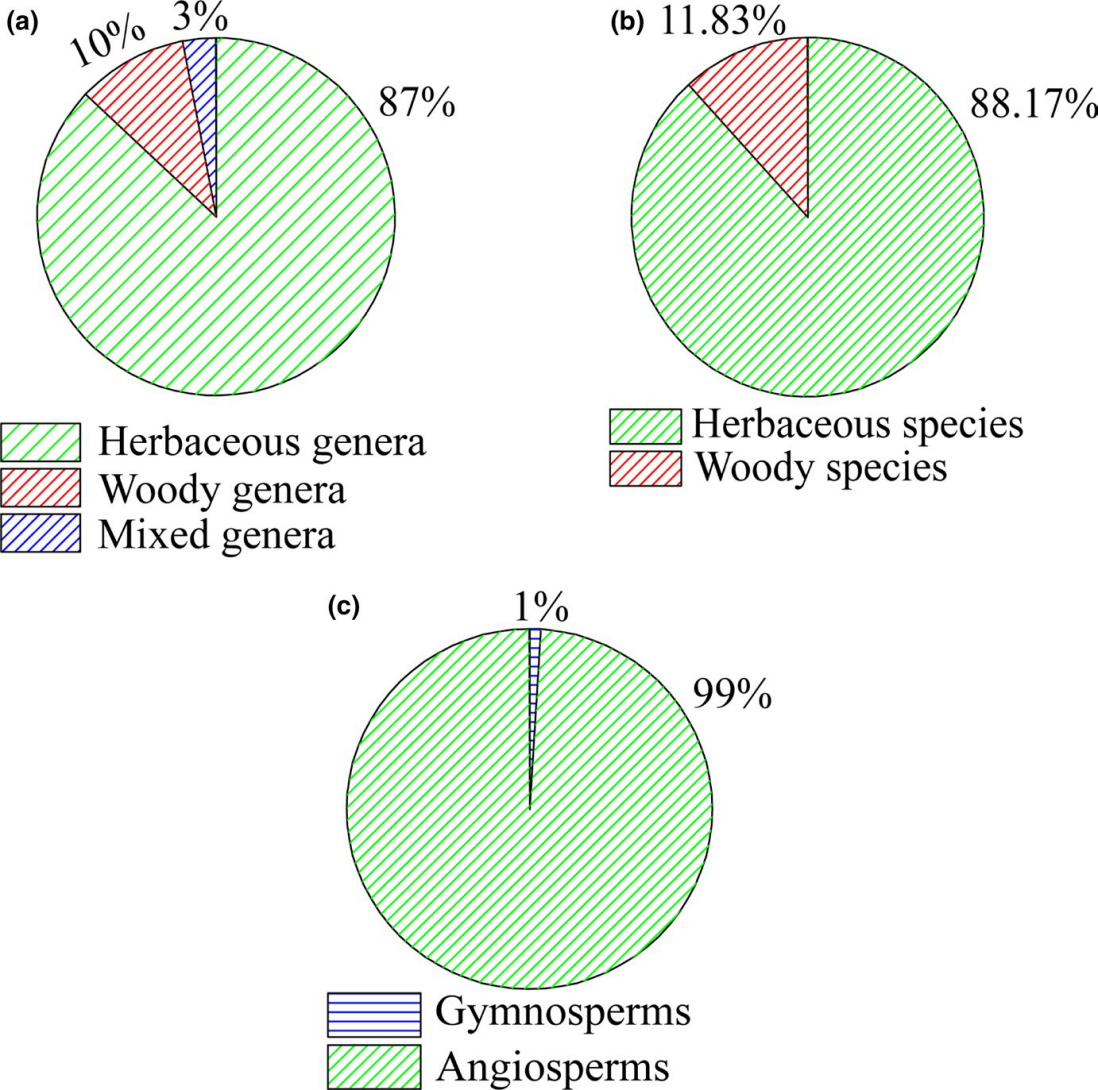
Taxonomic richness in the Kunlun Mountains. (a) genera richness of woody and herbaceous plants, (b) species richness of woody and herbaceous plants, and (c) gymnosperms and angiosperms of seed plants

The Kunlun Mountains flora is diverse with 1911 species (including subspecies and varieties), 397 genera, and 75 families. Gymnosperms accounted for only 26 of these seed plant species, which were further classified into five genera, three families, whereas angiosperms consisted of 1885 species (including subspecies and varieties), 392 genera, and 72 families (Figure 3c).

There were 226 woody species and 1685 herbaceous species in the Kunlun Mountains flora (Figure 3b). Specifically, the woody species consisted of 22 tree species, 197 shrub species, and seven liana species. The herbaceous species were represented by nine herbaceous climber species, 224 annual herb species, and 1,452 perennial herb species. At the genus level, these seed plants were spread across 347 herbaceous genera, 39 woody genera, and 11 mixed genera that included both woody and herbaceous species (Figure 3a). The plant species of 15 genera accounted for approximately one‐third of the total, the 15 genera included more than 20 species per genus. There were 155 monotypic genera.

**FIGURE 4:**
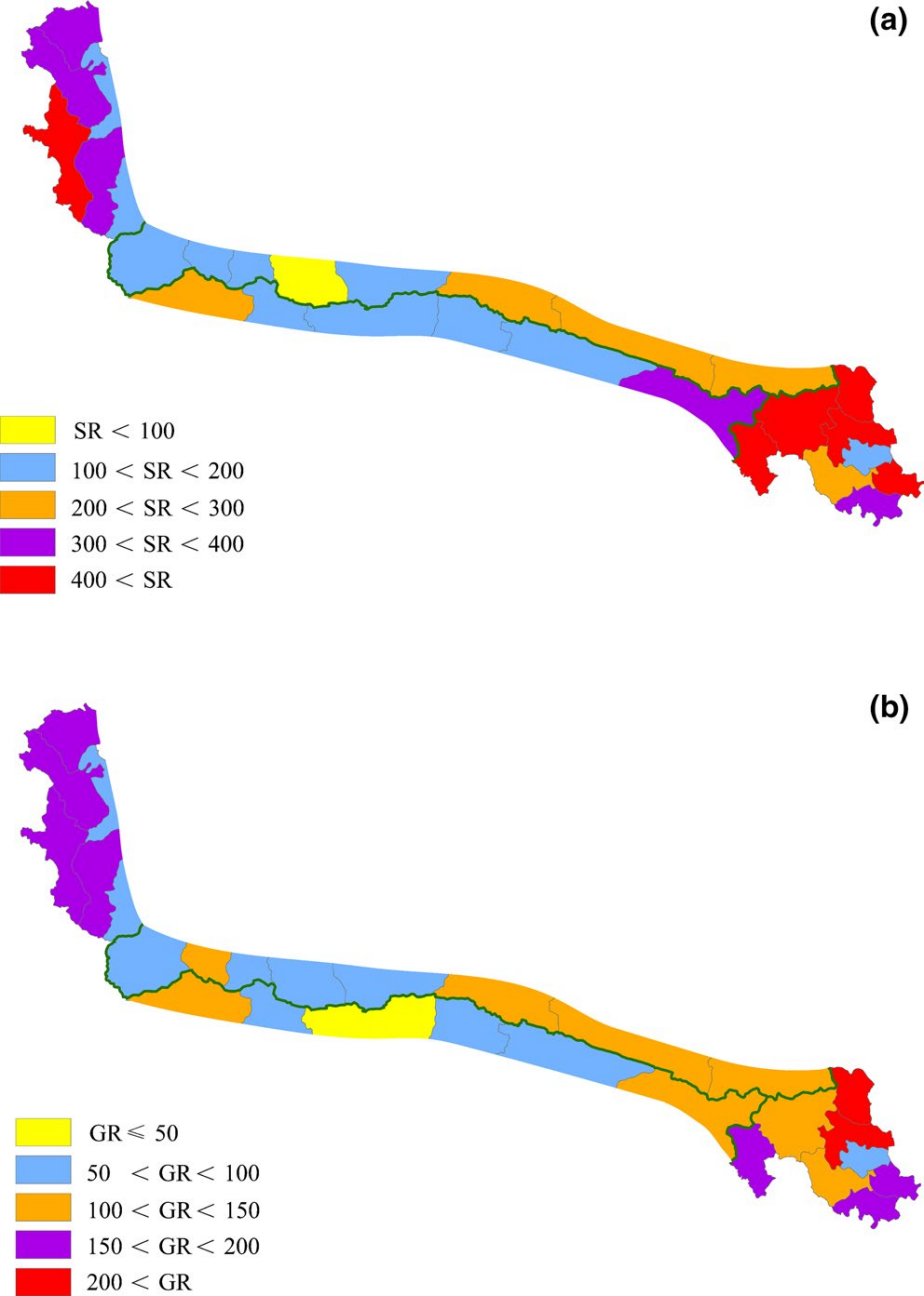
Patterns of species richness (SR) and genera richness (GR). (a) SR, and (b) GR

The gymnosperms found were all woody species and genera. The Pinaceae consisted of three genera, while the Ephedraceae and Cupressaceae were represented by one genus each. At the species/genus level, there were 18/5, 7/3, 4/2, and 9/3 gymnosperm species/genera in the eastern region, northern slopes of the central region, southern slopes of the central region, and western region of the Kunlun Mountains, respectively. Of the angiosperms, 200 were woody species, and 1685 were herbaceous species, with 34 woody genera, 347 herbaceous genera, and 11 mixed genera that included both woody and herbaceous species (Figure 3a). At the species/genus level, there were 1281/331, 632/195, 598/190, and 804/242 species/genera in the eastern region, northern slopes of the central region, southern slopes of the central region, and western region of the Kunlun Mountains, respectively. Therefore, the distribution pattern of species diversity was that the biodiversity in the south slope and north slope of the central regions were the lowest, and those in the eastern regions were observably higher than those in the western regions and central regions (Figure 4).

Seven genera were endemic to China, all herbaceous genera of angiosperms, and four only appeared in the eastern region of the Kunlun Mountains. There were three endemic genera in the eastern region and the central region of the Kunlun Mountains, and no endemic genera in the western region (Table 1). A total of 570 species were endemic to China, which accounted for 29.83% of the total species and included 489 herbaceous species and 81 woody species. The distribution pattern of these endemic species were 493, 129, 155, and 51 species in the eastern region, northern slopes of the central region, southern slopes of the central region, and western region of the Kunlun Mountains, respectively (Table 1).

Overall, the Kunlun Mountains flora was spatially varied (Table 1). The species and genera richness indicated that biodiversity in the eastern region was higher than those in the western and central regions (Table 1; Appendix S3). Similar results also characterized the endemic taxa on the Kunlun Mountains (Table 1). Consequently, the eastern region was the center of the species richness, genera richness, and endemic taxa in the Kunlun Mountains flora.

### 3.2 Geographical origin and divergence time of floras

In this study, 126 clades of seed plants (species or genus level) were collected, accounting for 126 genera, 55 families, and 30 orders of seed plants. There were five clades of gymnosperms and 121 clades of angiosperms. The five clades represented all gymnosperm species, while the 121 clades of angiosperms represented 61% of the species, 31% of the genera, 72% of the families, and 93% of the orders in angiosperms (Appendices S1 and S2).

Based on the vegetation types, there were 11 dominant genera and 50 common genera in the Kunlun Mountains, with 126 clades of seed plants including all dominant genera and 45 common genera (Appendices S1 and S2). The MDT of these clades could represent the divergence time of the Kunlun Mountains flora.

**FIGURE 5:**
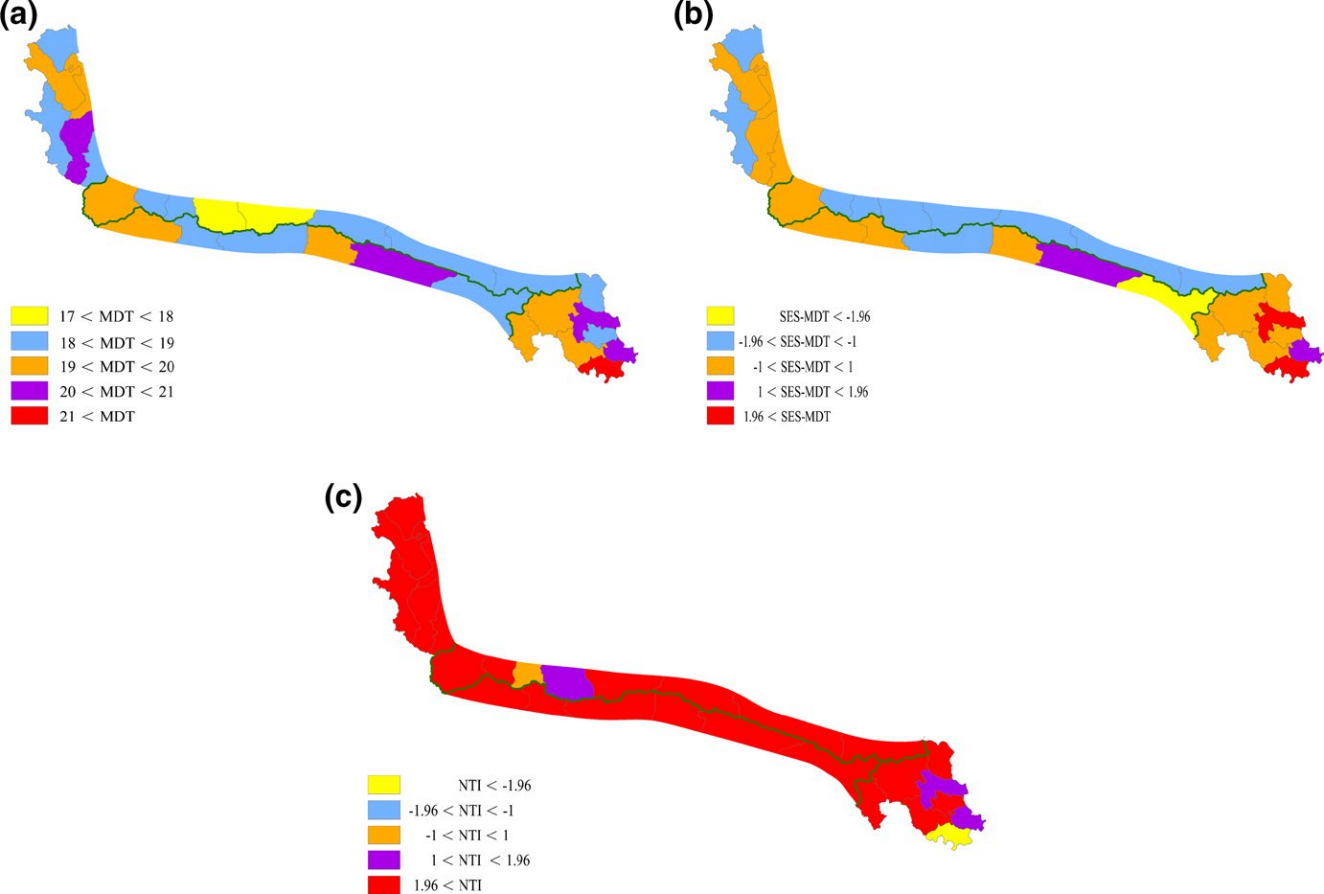
Patterns of mean divergence times (MDT), standardized effect size of the mean divergence time (SES‐MDT), and nearest taxon index (NTI) at the county‐level geographical units of the Kunlun Mountains. (a) MDT, (b) SES‐MDT, (c) NTI

The MDT of the Kunlun Mountains flora was in the early Miocene (19.40 Ma), and the median age of the Kunlun Mountains flora was in the middle Miocene (13.75 Ma). The MDT in the eastern, central, and western regions of the Kunlun Mountains was 20.07 Ma, 17.55 Ma, and 18.09 Ma, respectively. In addition, the MDT was 17.60 Ma and 17.18 Ma on the northern and southern slopes of the central Kunlun Mountains, respectively. The results showed that the eastern region was the center of distribution of ancient taxa in the Kunlun Mountains. Across the different county‐level geographical units, the maximum MDT was 22.77 Ma in Banma, and the minimum MDT was 17.28 Ma in Minfeng (Figure 5a). Two SES‐MDTs, namely, Banma and Maqin, were greater than 1.96, and only one SES‐MDT, namely, Qumalai, was <−1.96, with all showing significant differences (*p* < .05; Figure 5b). These significant SES‐MDTs revealed that the flora lineages were more ancient in Banma and Maqin (*p* < .05) and more recent in Qumalai (*p* < .05; Figure 5b). Overall, MDTs were greater at both ends of the areas, and the eastern flora was found to be older than the western flora (Figure 5a). However, the SES‐MDTs of the 24 counties did not show significant differences.

Geographical origin analysis of 126 clades on seed plants indicated that they were primarily from the Laurasian flora, such as Eastern Asia (40 clades), Tethyan (18 clades), and Northern Hemisphere unknown (28 clades), while only three clades were from the Gondwanan flora (Table 2; Table S1), which were only distributed in the eastern region.

**TABLE 2:** Geographical origin of 126 clades in the Kunlun Mountains

	Geographical origin	Clades
Laurasian floras (100 clades)	Eastern Asia	40
	Tethyan	18
	North America	3
	Southwest Asia	2, *Potentilla*, *Sibbaldia*
	Central Asia	3
	Eurasia	1, *Thalictrum*
	Southwest China	1, *Brachypodium*
	Northwest China	1, *Lagochilus*
	Qinghai‐Tibet plateau	1, *Pomatosace*
	Eastern Asia or Western North America	1, *Saxifraga*
	Central Asia or the Qinghai‐Tibet plateau	1, *Kengyilia*
	Northern Hemisphere unknown	28
Gondwanan floras (3 clades)	Africa	2, *Hypericum*, *Anaphalis*
	Southern Hemisphere unknown	1, *Nannoglottis*
Unknown (23 clades)	Unknown	23

### 3.3 Phylogenetic structure

The NTIs were calculated by the phylogenetic tree of angiosperms, and indicated that the counties had different phylogenetic structures (Figure 5c). In addition, the phylogenetic tree of gymnosperms could not calculate the index (Figure S1). The 27 NTIs were positive in county‐level communities, and 23 of these NTIs were statistically significant (*p* < .05). Furthermore, the only one NTI was negative in the Banma community and showed significant differences (*p* < .05; Figure 5c). Four counties, namely, Jiuzhi, Maqin, Minfeng, and Yutian, had positive NTIs but these were not statistically significant. Jiuzhi and Maqin are located in the southeastern region, while the other counties belong to the central regions and are adjacent to the Tarim Basin (Figure 2).

## 4 DISCUSSION

### 4.1 The patterns of the Kunlun Mountains flora

The Kunlun Mountains flora consisted of 1911 species, 397 genera, and 75 families. The gymnosperms were 26 species, five genera, and three families, the other taxa were all angiosperms. These taxa also belonged to 1685 herbaceous species and 226 woody species, which were divided into 347 herbaceous genera, 39 woody genera, and 11 mixed genera. There were 570 endemic species and seven endemic genera in the areas. Although the Kunlun Mountains are not considered a hotspot of Chinese endemic seed flora and a center of speciation for extant plants (Huang et al., 2012, 2016), we found a high proportion of Chinese endemic species in the Kunlun Mountains, which was similar to that in the Hengduan Mountains. Chinese endemic species accounted for approximately 20% of the total species in the QTP (Wu, 2008; Yan et al., 2013; Yu, Zhang, et al., 2018), and 32.4% of the total in the Hengduan Mountains (Zhang et al., 2009). The distribution pattern of taxa was that these taxa were mainly distributed in the eastern region of the Kunlun Mountains (Figure 4).

The results of MDT analysis indicate that the Kunlun Mountains flora is ancient (19.40 Ma) compared with the flora of western China (15.29–18.86 Ma; Lu et al., 2018). In addition, the median age of the Kunlun Mountains flora was estimated in the middle Miocene (13.75 Ma). Compared with other floras of the Northern Hemisphere (Chen et al., 2018), such as the formation times of the Californian flora (10.60 Ma; Baldwin, 2014), the East Asian flora (15.10 Ma; Chen et al., 2018), the Hengduan Mountains flora (13.60 Ma; Chen et al., 2018), the Kunlun Mountains flora was relatively ancient, and their median age was nearest between the Hengduan Mountains flora and the Kunlun Mountains flora. Furthermore, the MDT of the Kunlun Mountains flora in the eastern region was found to be greater than that in the central and western regions; therefore, the eastern region of Kunlun Mountains flora was the most ancient, which preserve abundant ancient taxa.

In county‐level geographical units, only one county‐level flora was older than 21 Ma, namely, Banma (22.53 Ma). There were four county‐level flora that diverged between 20 Ma and 21 Ma, namely, Jiuzhi (20.61 Ma), Maqin (20.97 Ma), Zhiduo (20.75 Ma), and Yecheng (20.13 Ma), while 21 county‐level flora had an MDT between 18 Ma and 20 Ma. The flora of Qiemo and Minfeng were less than 18 Ma, and the MDT was the youngest in Minfeng (17.28 Ma). In addition, three SES‐MDTs, namely, those of Banma, Maqin, and Qumalai, showed significant differences (*p* < .05; Figure 5b). The flora in Banma and Maqin were the most ancient. The MDT of the flora in Qumalai was 18.34 Ma, and the flora had more recent lineages (*p* < .05).

The results of geographical origin analyses indicated that the Kunlun Mountains flora was primarily from Eastern Asia (40 clades), Tethyan (18 clades), Northern Hemisphere unknown (28 clades), and Unknown (23 clades), while only one clade was from the QTP (Table 2). However, the MDT only represents temporal patterns of these clades in the Kunlun Mountains flora. Geographical origin of these clades revealed only their spatial patterns. The results of MDT indicated that the origin time of the Kunlun Mountains flora was determined to be later than the early Miocene (19.40 Ma) because temporal patterns of these clades could be assembled since 19.40 Ma. In addition, the geographical origin of these clades also indicates that spatial patterns of these clades are not in situ assemblies in the Kunlun Mountains. Therefore, the formation processes of the Kunlun Mountains flora could include at least *ex situ* speciation and dispersal since 19.40 Ma.

Recent studies have indicated that the formation of the QTP only occurred in the late Neogene (Spicer et al., 2020) and that the formation of the Asian monsoon system also emerged in the Neogene (Li et al., 2021; Xie et al., 2021). Some studies have recently demonstrated that the ecosystem of the QTP experienced a significant shift at the Paleogene/Neogene boundary (Deng et al., 2019) and that the Kunlun Mountains have reached their present height over the last 17 Ma (Pan, 2000; Sun et al., 2015). The arid climate of Central Asia appeared in the late Miocene, with an origin time of 5.3 Ma (i.e., 5.23–5.38 Ma), and multiple climate fluctuations have occurred since the early Pliocene, especially the Quaternary (Huang et al., 2015; Zhang et al., 2021; Zhang & Sun, 2011). Hence, the Kunlun Mountains did not rise to their present elevation in the early Pliocene (5.3 Ma). In addition, the divergence time of 103 clades was greater than 5.3 Ma, and the proportion of these clades was >80%. Previous studies have indicated that the vegetation types of the Kunlun Mountains were coniferous and broad‐leaved mixed forest of the warm temperate zone, and temperate deciduous forests and grasslands in the early Pliocene (5.3 Ma) and that the xerophytes have appeared in cotemporaneous flora (Huang, 1994). Therefore, a part of extant clades appeared in the Kunlun Mountains since 5.3 Ma. The extant Kunlun Mountains flora might have emerged in the early Pliocene (5.3 Ma).

Glacial events and dramatic climatic fluctuations have occurred in the Kunlun Mountains during the Quaternary (Deng et al., 2019; Owen et al., 2008; Owen & Dortch, 2014; Renner, 2016; Su, 1998), such as the Largest Glaciation (1.2–0.6 Ma) and the Last Glacial Maximum (Liu et al., 2014; Shi et al., 1997). Almost all species went extinct in the Kunlun Mountains because of the numerous glaciations. A recent study has highlighted that the main phylogeographical patterns of seed plant species in the Tibeto‐Himalayan region are contraction/recolonization, platform refugia/local expansion, and microrefugia (Ding et al., 2020; Muellner‐Riehl, 2019). However, some previous reports also indicated that few species, no Chinese endemic species included, were harbored in the platform refugia and microrefugia (López‐Pujol et al., 2011; Muellner‐Riehl, 2019). In addition, a recent study has suggested that no platform refugia existed on the Kunlun Mountains (Yu, Favre, et al., 2018). These studies indicated that the previous Kunlun Mountains flora was extinct during glacial periods of the Quaternary, the extant Kunlun Mountains flora were gradually formed during interglacial periods of the Quaternary. Consequently, after the climatic fluctuations and glaciations of the Quaternary, the extant biodiversity patterns might have dispersed from adjacent refugia, and primarily formed during the Quaternary (2.6 Ma).

Overall, the center of distribution of species diversity and endemic species were the eastern regions of the Kunlun Mountains. The MDT and the median age of the Kunlun Mountains flora were 19.40 Ma and 13.75 Ma, respectively. There were richly ancient taxa in the eastern region of Kunlun Mountains flora because the region was found to be the most ancient. The extant Kunlun Mountains flora might have emerged in the early Pliocene (5.3 Ma) according to geological evolution, uplift history, and paleovegetation in the areas. The action of climatic fluctuations and glaciations during the Quaternary has led to the relevant events, which were that the current species on the Kunlun Mountains were dispersed from adjacent refugia during the interglacial period of the Quaternary. Based on the floristic division of China, the Tethyan region and the QTP were separated by the Kunlun Mountains (Ye et al., 2019, 2020). Therefore, the biogeographical roles of the Kunlun Mountains are corridor and sink, and the corresponding key processes are species immigration and extinction. In addition, the Kunlun Mountains also represent a barrier and a boundary among the Tethyan region, the QTP, and East Asia.

### 4.2 The phylogenetic structure of the Kunlun Mountains flora

The extant biodiversity on the Kunlun Mountains occurred by species recolonization. A complex species recolonization was likely the most important evolutionary process to affect the deeper phylogenetic community structure. The evolutionary history of taxa was significant, particularly for the net relatedness index (Webb et al., 2002). NTI analyses can help reveal the phylogenetic structure in a community; NTIs primarily reveal the shallower parts of community phylogenetic structure. The complex sources of species colonization had little influence over NTIs. When NTI was closer to 0, the neutral theory could explain the community assembly. In contrast, niche theory may be used to reveal community assembly.

Based on the results of NTI, the community phylogenetic structure was dispersed in Banma, whereas the community phylogenetic structures were clustered in the other counties. Twenty‐four counties showed statistically significant NTIs (*p* < .05). The three positive NTI, namely, Jiuzhi, Maqin, and Minfeng, were greater than 1, and the NTI in Yutian was 0.97; however, they were not significantly different (Figure 5c).

Previous studies have revealed that abiotic determinism tends to increase with spatial scale, while biotic determinism tends to decrease with spatial scale. The abiotic determinism dominates biodiversity maintenance mechanisms at the regional scale; the biotic interactions had little effect on biodiversity (Cardillo, 2011; Charles et al., 2010; Niu et al., 2011; Villalobos et al., 2013; Yang et al., 2014). Therefore, the abiotic environment and evolutionary history of biodiversity patterns greatly influence the community phylogenetic structure at the regional scale (Kraft et al., 2007). In addition, in the QTP, the responses of species diversity to climate depend on the biotype. The diversity of woody plants was more strongly associated with climate than that of herbaceous plants. Energy and water availability jointly influence the diversity of woody plants, whereas water availability alone predominantly regulates the diversity of herbaceous plants (Yan et al., 2013). In the Kunlun Mountains, although the dominant vegetation type consists of herbaceous plants, a few coniferous forests are distributed in the eastern and western regions. Furthermore, annual precipitation tends to notably decrease from the east to the west (Wu, 2012–2015; Zheng, 1999), while there are numerous rivers in the eastern and western regions (Figure 2).

Therefore, the positive NTIs indicate that habitat filtering was primarily the driving force behind community assemblies. We speculate that water availability plays an important role in the current biodiversity pattern, particularly in the western and central regions of the Kunlun Mountains. Based on the vegetation type, forests were concentrated in Banma. The MDT of the flora in Banma was greater than 21 Ma, which was the most ancient flora. The combination of species from multiple floras and adequate hydrothermal conditions may explain the dispersion of phylogenetic structures in Banma.

## 5 CONCLUSIONS

The biodiversity patterns indicate that the eastern region of the Kunlun Mountains is a center of species richness and endemic taxa. However, compared with the flora in the southeastern part of the QTP, the Kunlun Mountains flora has relatively low biodiversity, which is consistent with the findings of previous studies (Lu et al., 2018; Mao et al., 2013; Yan et al., 2013).

The results of MDT analyses indicate that the divergence time of the Kunlun Mountains flora was in the early Miocene (19.40 Ma), and the eastern region was the most ancient. However, the extant biodiversity on the Kunlun Mountains appeared in the early Pliocene (5.3 Ma) and was formed by species recolonization during interglacial periods of the Quaternary. The Kunlun Mountains flora was primarily from Eastern Asia, Tethyan, Northern Hemisphere unknown, and unknown areas.

In conclusion, the biodiversity patterns were that the eastern regions were the center of species diversity, endemic species, and ancient taxa in the Kunlun Mountains. The current biogeographical roles of the Kunlun Mountains are corridor and sink, and the related key processes are species immigration and extinction. Habitat filtering was an important driving force for species migration into the Kunlun Mountains. In addition, the Kunlun Mountains also function as a barrier, representing a boundary among the Tethyan region, the QTP, and East Asia. The Kunlun Mountains have converted the QTP into a relatively closed geographical unit.

## CONFLICT OF INTEREST

The authors have no conflict of interest to declare.

## AUTHOR CONTRIBUTIONS

**Weibo Du:** Writing – original draft (lead); Writing – review & editing (lead). **Peng Jia:** Software (lead); Writing – review & editing (lead). **Guozhen Du:** Supervision (lead).

### Open Research Badges

This article has been awarded <Open Materials, Open Data, Preregistered> Badges. All materials and data are publicly accessible via the Open Science Framework at https://doi.org/10.5061/dryad.w0vt4b8sv. In addition, for private access during this review period, we may share our unpublished dataset using this temporary link: https://datadryad.org/stash/share/xGflo4kG9YIe502yvBVhWiwYk6KKitEqfB4Ky‐Rrr1w.

## Supporting information

Appendix S1

Appendix S2

Fig S1

Appendix S3

Appendix S4

Supplementary Material

## Data Availability

Data are available via the Dryad Digital Repository: https://doi.org/10.5061/dryad.w0vt4b8sv
