# Supplementary material for: Current biogeographical roles of the Kunlun Mountains
Source: Ecol Evol. 2022 Jan 15;12(1):e8493. doi: 10.1002/ece3.8493 (PMC8809438; doi:10.1002/ece3.8493)
Supplement: Supplementary file 4 — Appendix S3 [file ECE3-12-e8493-s005.docx]

**Supplementary Table 1.** Estimated divergence times and inferred geographical origins of seed plants clades in the Kunlun Mountains based on molecular studies.

| **Family** | **Genera** | **Species number** | **Geographical origin** | **Origin time** | **Note** | **Dating method** | **References** |
| --- | --- | --- | --- | --- | --- | --- | --- |
| **Ephedraceae** | *Ephedra* | 7 | Tethyan | 20（8-32）Ma | This time is the divergence time between the East Asian and Europe–Mediterranean Ephedra. | secondary calibration | 1-2 |
| **Pinaceae** | *Abies* | 2 | Northern Hemisphere unknown | 35.9 (22.9-51.8) Ma | This time is the divergence time between the East Asian and Europe–Mediterranean Abies. | fossil calibration | 3 |
|  | *Picea* | 7 | North America | 21.96 (12.52-36.38) Ma | This time is the crown age of the whole genus. | fossil calibration | 4 |
|  | *Larix* | 1 | Northern Hemisphere unknown | 85.62Ma | This time is the crown age of the the whole genus. | secondary calibration | 5-6 |
| **Cupressaceae** | *Juniperus* | 9 | Tethyan | 24.55 (19.3-29.8) Ma | This time is the crown age of the Juniperus sect. Sabina East Asian species were included in this section. | fossil calibration | 7 |
| **Alismataceae** | *Alisma* | 1 | Tethyan | 9.61 Ma | This time is the crown age of this genus. | fossil calibration | 8 |
| **Juncaginaceae** | *Triglochin* | 2 | Unknown | 39 Ma |  | secondary calibration | 9 |
| **Potamogetonaceae** | *Stuckenia* | 3 | Unknown | 13.49 Ma | This time is the diversification time of this genus. | secondary calibration | 10 |
| **Nartheciaceae** | *Aletris* | 1 | Eastern Asia | 34 Ma | This time is the crown age of this genus. | fossil calibration | 11 |
| **Liliaceae** | *Fritillaria* | 4 | Eastern Asia | 15.9 Ma | This time is the crown age of this genus. | fossil calibration | 12 |
|  | *Notholirion* | 1 | Eastern Asia | 26.45 (24.8-28.1) Ma | This time is the crown age of this genus. | fossil calibration | 12 |
| **Orchidaceae** | *Cypripedium* | 2 | Eastern Asia | 25 Ma | This time is the crown age of this genus. | secondary calibration | 13 |
| **Amaryllidaceae** | *Allium* | 19 | Northern Hemisphere unknown | 12.8Ma | This time is the diversification time of this genus. | secondary calibration | 14 |
| **Asparagaceae** | *Asparagus* | 3 | Unknown | 16.4 (8.6–25.0) Ma | This time is the crown age of this genus. | secondary calibration | 15 |
|  | *Polygonatum* | 2 | Unknown | 13.96 (14.34–13.57)Ma | This time is the crown age of this genus. | secondary calibration | 16 |
| **Juncaceae** | *Juncus* | 13 | Unknown | 69（87-51）Ma | This time is the origin time of this genus. | secondary calibration | 17 |
| **Cyperaceae** | *Carex* | 56 | Unknown | 36（41-34）Ma | This time is the origin time of this genus. | secondary calibration | 18 |
| **Poaceae** | *Stipa* | 25 | Northern Hemisphere unknown | 5.8（9-2.61）Ma | This time is the diversification time of this genus. | secondary calibration | 19 |
|  | *Kengyilia* | 12 | Central Asia or the Qinghai-Tibet plateau | 4.92 (2.72–7.12) Ma | This time is the crown age of this genus. | secondary calibration | 20 |
|  | *Agropyron* | 1 | Central Asia | 1.08 Ma | This time is the diversification time of this genus. | secondary calibration | 20 |
|  | *Brachypodium* | 1 | Southwest China | 3 Ma | This time is the crown age of this genus. | secondary calibration | 21 |
|  | *Hordeum* | 3 | Tethyan | 5.3 (4.5-6.1) Ma | This time is the divergence time between the East Asian Hordeum and its close relatives. | secondary calibration | 22 |
|  | *Leymus* | 12 | Tethyan | 11.5(11-12)Ma  4(3.7-4.3) Ma,1.9(1.7-2.1) Ma | This time is the crown age of this genus.  This time is the diversification time of this genus. | secondary calibration | 23 |
|  | *Anthoxanthum* | 2 | Unknown | 17.45(22.6-12.3) Ma (plastid)  25.5(30-21) Ma (nuclear) | This time is the crown age of this genus. | secondary calibration | 24 |
|  | *Festuca* | 18 | Unknown | 12 Ma | This time is the crown age of this genus. | secondary calibration | 25 |
|  | *Puccinellia* | 30 | Unknown | 2Ma | This time is the quickly diversification time of this genus. | secondary calibration | 26-27 |
|  | *Poa* | 34 | Unknown | 13.75(9.9-17.6) Ma | This time is the crown age of this genus. | secondary calibration | 26-27 |
|  | *Aristida* | 1 | Unknown | 7.5 (4.4–10.6) Ma | This time is the crown age of the genus. | fossil calibration | 28 |
| **Papaveraceae** | *Meconopsis* | 7 | Eastern Asia | 16.6 Ma | This time is the divergence time between this genus and its close relatives. | substitution rate | 29 |
|  | *Corydalis* | 19 | Unknown | 28.47(31.37–25.56) Ma | This time is the crown age of the genus. | secondary calibration | 30 |
| **Berberidaceae** | *Berberis* | 7 | Northern Hemisphere unknown | 17(28-6) Ma | This time is the crown age of this genus. | secondary calibration | 31 |
|  | *Sinopodophyllum* | 1 | Eastern Asia | 5.8 (5.2-6.4) Ma | This time is the divergence time between Sinopodophyllum and its North American relative Podophyllum. | secondary calibration | 32 |
| **Ranunculaceae** | *Caltha* | 2 | Northern Hemisphere unknown | 39.7 (27.7-53.8) Ma | This time is the divergence time between two clades. And East Asian species were included in caldeⅠ.] | fossil calibration | 33 |
|  | *Aconitum* | 7 | Northern Hemisphere unknown | 12.68 Ma | This time is the crown time of the whole Aconitum. | secondary calibration | 34 |
|  | *Delphinium* | 15 | Northern Hemisphere unknown | 5.8 Ma | This time is the crown age Delphinium. | secondary calibration | 34 |
|  | *Aquilegia* | 1 | Eastern Asia | 4.8 Ma | This time is the divergence time between this genus and its close relatives. | fossil calibration | 35 |
|  | *Thalictrum* | 8 | Eurasia | 8.2(5.8-10.6) Ma | This time is the crown age of Thalictrum | fossil calibration | 36-37 |
|  | *Clematis* | 5 | Northern Hemisphere unknown | 7.81(3.99-13.14) Ma | This time is the diversification time of the whole genus. | fossil calibration | 38 |
|  | *Ranunculus* | 18 | Northern Hemisphere unknown | 23.7 Ma | This time is the crown age of acris clade and Eurasia was proposed as the ancestral area for this clade. | fossil calibration | 39 |
| **Saxifragaceae** | *Saxifraga* | 28 | Eastern Asia or Western North America | 30.51(23.87-37.15)Ma | This time is the divergence time between this genus and its close relatives. | secondary calibration | 40 |
| **Crassulaceae** | *Rhodiola* | 18 | Eastern Asia | 21.02 (10.7-36.1) Ma | This time is the diversification time of the Rhodiola, Pseudosedum and its close relative. This study also suggested that merging Pseudosedum with Rhodiola. | fossil calibration | 41 |
| **Zygophyllaceae** | *Zygophyllum* | 7 | Northern Hemisphere unknown | 19.56 Ma（11.25-28.78Ma） | This time is the rapid differentiation within genera. | secondary calibration | 42 |
| **Fabaceae** | *Thermopsis* | 5 | Eastern Asia | 0.97 (0.46-2.8) Ma | This time is the divergence time between this genus and its close relatives. | fossil calibration | 43 |
|  | *Caragana* | 16 | Tethyan | 15 (14-16) Ma | This time is the crown age of this genus. | fossil calibration | 44-45 |
|  | *Phyllolobium* | 3 | Eastern Asia | 8.48 (6.67-10.28) Ma | This time is the divergence time between Phyllolobium and its relative Swainsona. Phyllolobium is a recently erected genus from subgenus Pogonophace of Astragalus. | fossil calibration | 46 |
|  | *Astragalus* | 83 | Unknown | 18.45(16.1-20.8) Ma | This time is the divergence time between this genus and its close relatives. | secondary calibration | 47 |
|  | *Oxytropis* | 54 | Unknown | 18.45(16.1-20.8) Ma | This time is the divergence time between this genus and its close relatives. | secondary calibration | 47 |
|  | *Tibetia* | 1 | Eastern Asia | 15.23 (7.27-25.18) Ma | This time is the divergence time between this genus and its close relatives. | secondary calibration | 49 |
|  | *Hedysarum* | 8 | Tethyan | 15.46(9.79-21.13) Ma | This time is the divergence time between this genus and its close relatives. | secondary calibration | 50 |
| **Rosaceae** | *Spiraea* | 7 | Northern Hemisphere unknown | 13.4 Ma | This time is the diversification time of this genus. | secondary calibration | 51-52 |
|  | *Sibiraea* | 2 | Northern Hemisphere unknown | 3.70Ma | This time is the diversification time of this genus. | secondary calibration | 51-53 |
|  | *Cotoneaster* | 13 | Northern Hemisphere unknown | 30.5(22-39)Ma | [This time is the crown age of the whole genus.] | secondary calibration | 54 |
|  | *Sorbus* | 3 | Northern Hemisphere unknown | 32(28-36) Ma | This time is the crown age of the whole genus. | secondary calibration | 54 |
|  | *Malus* | 2 | Eastern Asia | 33.92(19.44-45.13)Ma | This time is the crown age of the whole genus. | secondary calibration | 55 |
|  | *Dasiphora* | 8 | Northern Hemisphere unknown | 23.4 (14.6-32.9) Ma | This time is the crown age of the whole genus. | secondary calibration | 56-57 |
|  | *Potentilla* | 33 | Southwest Asia | 20.65 (16.7-24.6) Ma | This time is the crown age of the genus. | fossil calibration | 58-59 |
|  | *Sibbaldia* | 4 | Southwest Asia | 19.4(12.4-26.8) Ma | This time is the crown age of the genus. | secondary calibration | 59 |
|  | *Rosa* | 10 | Northern Hemisphere unknown | 40.1(30.0-50.2) Ma | The age is the first divergence event in Rosa | secondary calibration | 60 |
|  | *Cerasus* | 3 | Eastern Asia | 61 Ma | This time is the appeared time for modern Prurus. | fossil calibration | 61 |
| **Elaeagnaceae** | *Hippophae* | 4 | Central Asia | 5.46 Ma（2.23-10.10 Ma） | This time is the crown age of the whole genus. | secondary calibration | 62 |
| **Urticaceae** | *Urtica* | 4 | Unknown | 26.2（18.7-33.8）Ma | This time is the crown age of this genus. | secondary calibration | 63 |
|  | *Parietaria* | 1 | Unknown | 14.7（5.4-24.6）Ma | This time is the crown age of this genus. | secondary calibration | 63 |
| **Betulaceae** | *Betula* | 3 | Northern Hemisphere unknown | 49 (60–38) Ma | This time is the crown age of this genus. | fossil calibration | 64 |
| **Hypericaceae** | *Hypericum* | 1 | Africa | 24.39 (19.61-19.19) Ma | This time is the divergence time between the clade D (included most species of East Asia) and clade E. The Old -World Hypericum contains three clades: C, D, E. | fossil calibration | 65 |
| **Violaceae** | *Viola iwagawae-tashiroi species complex* | 5 | Eastern Asia | 3.17 (1.37-4.93) Ma | This time is the crown age of this complex. | secondary calibration | 66 |
| **Salicaceae** | *Salix* | 33 | Northern Hemisphere unknown | 43.87 (37.15-48.42) Ma | This time is the crown age of the whole genus. | fossil calibration | 67 |
| **Geraniaceae** | *Geranium* | 5 | Tethyan | 10Ma  5Ma | This time is the crown age of this genus.  This time is the diversification time of this genus. | secondary calibration | 68 |
| **Onagraceae** | *Circaea* | 1 | North America | 16.17 (7.69-24.53) Ma | This time is the crown age of the whole genus. | fossil calibration | 69 |
| **Nitrariaceae** | *Nitraria* | 3 | Tethyan | 9 Ma | This time is the diversification time of this genus. | fossil calibration | 70 |
| **Thymelaeaceae** | *Stellera* | 1 | Eastern Asia | 2.38 Ma | This time is the diversification time of this genus. | substitution rate | 71 |
| **Brassicaceae** | *Capsella* | 1 | Unknown | 10.5(8.8-12.2)Ma | This time is the origin time of the genus. | secondary calibration | 72 |
|  | *Draba* | 19 | Tethyan | 2.04 (1.36–2.71) Ma | This time is the origin time of the genus. | substitution rate | 73 |
|  | *Crucihimalaya* | 1 | Unknown | 10.5(8.8-12.2)Ma | This time is the origin time of the genus. | secondary calibration | 72 |
|  | *Dontostemon* | 3 | Tethyan | 11.71 (7.12-16.83) Ma | This time is the crown age of this genus. | substitution rate | 74 |
|  | *Solms-laubachia* | 2 | Tethyan | 2.31 (1.42-3.68) Ma | This time is the stem age of the Solms-laubachia in the Hengduan Mountains and is the crown age for the whole genus | fossil calibration | 75 |
| **Tamaricaceae** | *Reaumuria* | 2 | western Central Asia | 19.78(7.52-37.52) Ma | This time is the crown age of whole genus. | secondary calibration | 76 |
|  | *Myricaria* | 6 | Eastern Asia | 20 Ma | This time is the crown age of whole genus. | fossil calibration | 77 |
| **Plumbaginaceae** | *Limonium* | 5 | Tethyan | 10 Ma | This time is the crown age of whole genus. | fossil calibration | 78 |
| **Polygonaceae** | *Koenigia* | 9 | Eastern Asia | 8.89 (4.82-13.58) Ma | This time is the divergence time between Koenigia and its close relatives. | fossil calibration | 79 |
|  | *Rumex* | 6 | Northern Hemisphere unknown | 15Ma | This time is the diversification time of this genus. | secondary calibration | 80 |
|  | *Rheum* | 10 | Eastern Asia | 12 (8.2-16.1) Ma | This time is the diversification time of this genus. | fossil calibration | 81 |
| **Caryophyllaceae** | *Silene* | 10 | Unknown | 16Ma | This time is the crown age of the whole genus. | secondary calibration | 82-83 |
|  | *Dianthus* | 2 | Tethyan | 4.45 (1.9-7.0) Ma | This time is the diversification time of this genus. | fossil calibration | 84 |
| **Hydrangeaceae** | *Philadelphus* | 1 | North America | 18.8 Ma | This time is the crown age of the core Philadelphus. | fossil calibration | 85 |
| **Balsaminaceae** | *Impatiens* | 1 | Eastern Asia | 22.5 (16.9-28.1) Ma | This time is the crown age of the whole genus. | fossil calibration | 86 |
| **Primulaceae** | *Primula* | 20 | Unknown | 25Ma  8Ma | This time is the crown age of the whole genus.  This time is the diversification time of this genus. | secondary calibration | 87-89 |
|  | *Androsace* | 17 | Northern Hemisphere unknown | 18.02Ma | This time is the crown age of the whole genus. | secondary calibration | 90 |
|  | *Pomatosace* | 1 | Qinghai-Tibet Plateau | 1.16(1.39-0.92)Ma | This time is the crown age of the whole genus. |  | 91 |
| **Ericaceae** | *Rhododendron* | 8 | Eastern Asia | 24.37Ma | This time is the origin time of the genus across the Northern Hemisphere. | fossil calibration | 92 |
| **Gentianaceae** | *Gentiana* | 45 | Eastern Asia | 29.5 (21-38) Ma | This time is the crown age of whole genus. | fossil calibration | 93 |
|  | *Halenia* | 1 | Eastern Asia | 6.79(4.19 -9.38) Ma | This time is the divergence time between East Asian species Halenia elliptica and remainder species of this genus. | substitution rate | 94 |
| **Boraginaceae** | *Microula* | 14 | Eastern Asia (Hengduan Mountainous Region) | 19(20.5-17.5)Ma  2(3.4-0.6)Ma | This time is the crown age of whole genus.  This time is the diversification time of this genus. | secondary calibration | 95 |
| **Convolvulaceae** | *Convolvulus* | 4 | Tethyan | 17.9 (11.8–23.7) Ma. | This time is the crown age of whole genus. | secondary calibration | 96 |
| **Solanaceae** | *Physochlaina* | 1 | Northern Hemisphere unknown | 10 Ma | This time is the crown age of whole genus. | fossil calibration | 97 |
|  | *Hyoscyamus* | 1 | Unknown | 10 Ma | This time is the crown age of whole genus. | fossil calibration | 98 |
|  | *Anisodus* | 1 | Eastern Asia | 9.3 Ma | This time is the divergence time between this genus and its relatives. | fossil calibration | 98 |
|  | *Przewalskia* | 1 | Eastern Asia | 5.75 (2.35-9.44) Ma | This time is the divergence time between this genus and its relatives. | fossil calibration | 98 |
|  | *Mandragora* | 1 | Tethyan | 9.82 (4.40-16.18) Ma | This time is the divergence time between the East Asian species and its European relatives. | fossil calibration | 98 |
| **Plantaginaceae** | *Lagotis* | 8 | Eastern Asia | 19.3 (11.3-29.6) Ma | This time is the divergence time between Lagotis and its close relative Wulfenia. | fossil calibration | 99 |
|  | *Hippuris* | 1 | Unknown | 0.48 Ma | This time is the diversification time of this genus. | secondary calibration | 100 |
| **Bignoniaceae** | *Incarvillea* | 4 | Eastern Asia (Hengduan Mountainous Region) | 4.4Ma | This time is the diversification time of this genus. | secondary calibration | 101 |
| **Labiatae** | *Eriophyton* | 2 | Unknown | 1.28 Ma | This time is the diversification time of this genus. | secondary calibration | 102 |
|  | *Lagochilus* | 3 | Northwest China | 2.45(2.1-2.8)Ma | This time is the diversification time of this genus. | secondary calibration | 103 |
|  | *Isodon* | 1 | Eastern Asia | 27.32 (17.03-39.84) Ma | This time is the divergence time between this genus and its close relatives. | fossil calibration | 104 |
| **Mazaceae** | *Lancea* | 1 | Eastern Asia | 8.63Mya | This time is the divergence time between this genus and its relatives. | secondary calibration | 105 |
| **Orobanchaceae** | *Euphrasia* | 2 | Northern Hemisphere unknown | 9 (7-11) Ma | This is the crown age of this genus. | fossil calibration | 106 |
|  | *Pedicularis* | 54 | Northern Hemisphere unknown | 25.13(11.7-38.56) Ma | This is the crown age of this genus. | fossil calibration | 107-108 |
| **Campanulaceae** | *Cyananthus* | 1 | Eastern Asia | 15.12 (12.12-22.94) Ma | This time is the divergence time between Cyananthus and its close relatives the Codonopsis–Leptocodon clade. | fossil calibration | 109 |
| **Asteraceae** | *Nannoglottis* | 3 | Southern Hemisphere unknown | 27.5 (23-32) Ma | This time is the divergence time between Nannoglottis and other taxon of Asteraceae. | substitution rate | 110 |
|  | *Leontopodium* | 11 | Tehtyan | 10.31 (7.59-13.59) Ma | This time is the divergence time between this genus and its close relatives. | fossil calibration | 111 |
|  | *Anaphalis* | 7 | Africa | 7.04 (5.45-8.89) Ma | This time is the divergence time between this genus and its close relatives. | fossil calibration | 111 |
|  | *Artemisia* | 46 | Northern Hemisphere unknown | 19.8 (17.5-22.1) Ma | This time is the crown age of Artemisia group. | fossil calibration | 112 |
|  | *Parasenecio* | 1 | Eastern Asia | 11.29(2.58-20)Ma | This time is the divergence time between this genus and its close relatives. | fossil calibration | 113 |
|  | *Ligularia* | 12 | Eastern Asia | 11.29(2.58-20)Ma | This time is the divergence time between this genus and its close relatives. | fossil calibration | 113 |
|  | *Cremanthodium* | 9 | Eastern Asia | 11.29(2.58-20)Ma | This time is the divergence time between this genus and its close relatives. | fossil calibration | 113 |
|  | *Saussurea* | 65 | Eastern Asia | 10.5 (7-14) Ma | This time is the crown age of whole genus. | fossil calibration | 114 |
|  | *Xanthopappus* | 1 | Eastern Asia | 5.2 (4.7-5.7) Ma | This time is the divergence time between this genus and its relatives. | fossil calibration | 115 |
|  | *Tragopogon* | 2 | Tethyan | 2.6 (1.7-5.4) Ma | This time is the crown of this genus. | fossil calibration | 116 |
|  | *Soroseris* | 2 | Eastern Asia | 4.31 (3.79-4.82) Ma | This time is the divergence time between this genus and its close relatives. The author suggest merge Stebbinsia with Soroseris as Soroseris sect. Dubyaeopsis. | fossil calibration | 117-118 |
|  | *Syncalathium* | 1 | Eastern Asia | 4.93 (4.35-5.5) Ma | This time is the divergence time between this taxon and its close relatives | fossil calibration | 117-118 |
| **Caprifoliaceae** | *Lonicera Asian clade* | 11 | Northern Hemisphere unknown | 12 (7-17) Ma | This time is the origin time of the genus across the Northern Hemisphere. | secondary calibration | 119 |
| **Araliaceae** | *Eleutherococcus* | 2 | Eastern Asia | 25Ma | This time is the origin time of the genus across the Northern Hemisphere. | secondary calibration | 120 |
| **Apiaceae** | *Pleurospermum* | 9 | Northern Hemisphere unknown | 33.70Ma | This time is the crown age of the genus. | secondary calibration | 121-122 |
|  | *Bupleurum* | 6 | Northern Hemisphere unknown | 33.70Ma | This time is the crown age of the genus. | secondary calibration | 121-122 |
|  | *Notopterygium* | 2 | Eastern Asia | 4.03(1.74-6.32) Ma | This time is the crown age of the genus. | secondary calibration | 123 |
|  | *Angelica* | 2 | Eastern Asia | 13.6 (11.7-15.6) Ma | This time is the crown age of the Angelica group. | secondary calibration | 124 |

**Acknowledgments**

We thank the websites which have conducted extensive research on the phylogeography, systematics, and evolution. The websites: <http://www.timetree.org/>, <http://www.mobot.org/MOBOT/research/APweb/>

**References**

1.Yang Yong (2002) Systematics and Evolution of *Ephedra* L.（Ephedraceae）from China. Institute of Botany, The Chinese Academy of Sciences. Beijing.

2.Liu Haiming (2005) Fossil Records of *Ephedra* and its early Diversity. Institute of Botany, The Chinese Academy of Sciences. Beijing.

3.Xiang QP, Wei R, Shao YZ, et al (2015) Phylogenetic relationships, possible ancient hybridization, and biogeographic history of *Abies* (Pinaceae) based on data from nuclear, plastid, and mitochondrial genomes. Molecular Phylogenetics and Evolution. 82: 1–14.

4.Ran JH, Shen TT, Liu WJ, et al (2015) Mitochondrial introgression and complex biogeographic history of the genus *Picea*. Molecular Phylogenetics and Evolution. 93: 63–76.

5.Ran Jin-Hua, Shen Ting-Ting, Wua Hui, Gong Xun, Wang Xiao-Quan (2018) Phylogeny and evolutionary history of Pinaceae updated by transcriptomic analysis. Molecular Phylogenetics and Evolution. 129: 106–116.

6.Wei Xiaoxin (2004) Molecular evolution and biogeography of *Larix* (Pinaceae). Institute of Botany, The Chinese Academy of Sciences. Beijing.

7.Mao K, Hao G, Liu J, et al (2010) Diversification and biogeography of *Juniperus* (Cupressaceae): variable diversification rates and multiple intercontinental dispersals. New Phytologist. 188(1): 254–272.

8.Chen LY, Chen JM, Gituru RW, et al (2012) Generic phylogeny and historical biogeography of Alismataceae, inferred from multiple DNA sequences. Molecular Phylogenetics and Evolution. 63 (2): 407–416.

9.Von Mering Sabine, Kadereit Joachim W (2015) Phylogeny, biogeography and evolution of *Triglochin* L. (Juncaginaceae)--morphological diversification is linked to habitat shifts rather than to genetic diversification. Molecular Phylogenetics and Evolution.83: 200–212.

10.Du Zhi-Yuan, Wang Qing-Feng (2016) Allopatric divergence of *Stuckenia filiformis* (Potamogetonaceae) on the Qinghai-Tibet Plateau and its comparative phylogeography with *S. pectinata* in China. Scientific Reports. 6: 1–10.

11.Zhao Yimin (2009) Systematics and Biogeography of *Aletris* L. (Nartheciaceae). University of Chinese Academy of Sciences. Beijing.

12.Givnish TJ, Zuluaga A, Marques I, et al (2016) Phylogenomics and historical biogeography of the monocot order Liliales: out of Australia and through Antarctica. Cladistics. 32.

13.Li Jihong (2010) Molecular systematics and Biogeography of *Cypripedium* L. University of Chinese Academy of Sciences. Beijing.

14.Hauenschild F, Favre A, Schnitzler J, Michalak I, Freiberg M, Muellner-Riehl AN (2017) Spatio-temporal evolution of *Allium* L. in the Qinghai-Tibet-Plateau region: Immigration and in situ radiation. Plant Diversity. 39(4):167–179.

15.Chen Shichao, Kim Dong-Kap, Chase Mark W., Kim Joo-Hwan (2013) Networks in a Large-Scale Phylogenetic Analysis:Reconstructing Evolutionary History of Asparagales(Lilianae) Based on Four Plastid Genes. Plos one.8 (3): 1–18

16.Wang Jia-Jian, Yang Yong-Ping, Sun Hang, Wen Jun, Deng Tao, Nie Ze-Long, Meng Ying (2016) The Biogeographic South-North Divide of Polygonatum (Asparagaceae Tribe Polygonateae) within Eastern Asia and Its Recent Dispersals in the Northern Hemisphere. PloS one.11: 1–15.

17.Yanis Bouchenak-khelladi, A. Muthama Muthama, H. Peter Linder (2014) A revised evolutionary history of Poales:origins and diversification. Botanical Journal of the Linnean Society. 175: 4–16.

18.Léveillé-Bourret É, Starr JR, Ford BA (2018) Why are there so many sedges? Sumatroscirpeae, a missing piece in the evolutionary puzzle of the giant genus Carex (Cyperaceae). Molecular Phylogenetics and Evolution. 119: 93–104.

19.Chen Junjun (2017) Study on the evlution history of Chinese Grassland based on phylogeny of Stipa. Inner Mongolia University. Huhhot.

20.Xing Fan, Li-Na Sha, Jian Zeng, Hou-Yang Kang, Hai-Qin Zhang, Xiao-Li Wang, Li Zhang, Rui-Wu Yang, Chun-Bang Ding, You-Liang Zheng, Yong-Hong Zhou (2012) Evolutionary Dynamics of the Pgk1 Gene in the Polyploid Genus *Kengyilia* (Triticeae: Poaceae) and Its Diploid Relatives. PloS one. 7 (2): e31122.

21.Wu Popo (2018) Phylogeny and Biogeography Studies of Chinese Brachypodiaea. Shandong Normal University. Jinan.

22.Blattner FR. (2006) Multiple intercontinental dispersals shaped the distribution area of *Hordeum* (Poaceae). New Phytologist. 169(3): 603–614.

23.Fan X, Sha LN, Yang RW, Zhang HQ, Kang HY, Ding CB, Zhang L, Zheng YL, Zhou YH. (2009) Phylogeny and evolutionary history of *Leymus* (Triticeae; Poaceae) based on a single-copy nuclear gene encoding plastid acetyl-CoA carboxylase. BMC Evolutionary Biology. 9:1–15.

24.Manuel Pimentel, Elvira Sahuquillo, Zeltia Torrecilla, Magnus Popp, Pilar Catala´n, Christian Brochmann (2013) Hybridization and long-distance colonization at different time scales: towards resolution of long-term controversies in the sweet vernal grasses (*Anthoxanthum*). Annals of Botany. 112: 1015–1030.

25.Luis A. Inda, Isabel Sanmartín, Sven Buerki, Pilar Catalán (2014) Mediterranean origin and Miocene-Holocene old world diversification of meadow fescues and ryegrasses (Festuca subgenus Schedonorus and Lolium). Journal of Biogeography. 41 (3): 600–614.

26.Matthias H. Hoffmann, Julia Schneider, Philipp Hase, Martin Ro¨ser (2013) Rapid and Recent World-Wide Diversification of Bluegrasses (Poa, Poaceae) and Related Genera. Plos One. 8 (3): e60061.

27.Rosa Cerros-Tlatilpa, J. Travis Columbus, Nigel P. Barker (2011) Phylogenetic relationships of *Aristida* and relatives (Poaceae, Aristidoideae) based on noncoding chloroplast (trnL‐F, rpl16) and nuclear (ITS) DNA sequences. American Journal of Botany. 98(11): 1868–1886

28.Liliana M. Giussani, Lynn J. Gillespie, M. Amalia Scataglini, Marıa A. Negritto, M. Anton, Robert J. Soreng (2016) Breeding system diversification and evolution in American Poa supersect.Homalopoa (Poaceae: Poeae: Poinae). Annals of Botany. 118: 281–303.

29.Xie H, Ash JE, Linde CC, et al. (2014) Himalayan-Tibetan plateau uplift drives divergence of polyploid poppies: Meconopsis viguier (Papaveraceae). PloS one. 9 (6): e99177.

30.Miguel A. Pérez-Gutiérrez, Ana T. Romero-García, M. Carmen Fernández, G. Blanca, María J. Salinas-Bonillo, Víctor N. Suárez-Santiago (2015) Evolutionary history of fumitories (subfamily Fumarioideae,Papaveraceae): An old story shaped by the main geological and climatic events in the Northern Hemisphere. Molecular Phylogenetics and Evolution. 88: 75–92.

31.Yanxia Sun, Michael J.Moore, Jacob B.Landis, Nan Lin, Li Chen, TaoDeng, Jianwen Zhang, Aiping Meng, Shoujun Zhang, Komiljon Sh.Tojibaev, HangSun, Hengchang Wang (2018) Plastome phylogenomics of the early-diverging eudicot family Berberidaceae. Molecular phylogenetics and evolution. 128, 203–211.

32.Wang W, Chen ZD, Liu Y, et al (2007) Phylogenetic and biogeographic diversification of Berberidaceae in the northern hemisphere. Systematic Botany. 32(4): 731–742.

33.Cheng J, Xie L (2014) Molecular phylogeny and historical biogeography of *Caltha* (Ranunculaceae) based on analyses of multiple nuclear and plastid sequences. Journal of Systematics and Evolution. 52 (1): 51–67.

34.Jabbour, F, Renner, SS (2012) A phylogeny of Delphinieae (Ranunculaceae) shows that Aconitum is nested within Delphinium and that Late Miocene transitions to long life cycles in the Himalayas and Southwest China coincide with bursts in diversification. Molecular Phylogenetics and Evolution. 62 (3): 928–942.

35.Fior S, Li M, Oxelman B, et al. (2013) Spatiotemporal reconstruction of the Aquilegia rapid radiation through next-generation sequencing of rapidly evolving cpDNA regions. New Phytologist. 198 (2): 579–92.

36.Valerie L. Soza, Johanne Brunet, Aaron Liston, Patricia Salles Smith, Verónica S. Di Stilio (2012) Phylogenetic insights into the correlates of dioecy in meadow-rues (Thalictrum, Ranunculaceae). Molecular Phylogenetics and Evolution. 63: 180–192.

37.Valerie L. Soza, Kendall L. Haworth, Vero´nica S. Di Stilio (2013) Timing and Consequences of Recurrent Polyploidy in Meadow-Rues (Thalictrum, Ranunculaceae). Molecular Biology and Evolution. 30 (8):1940–1954

38.Xie L, Wen J, Li LQ (2011) Phylogenetic analyses of *Clematis* (Ranunculaceae) based on sequences of nuclear ribosomal ITS and three plastid regions. Systematic Botany. 36(4): 907–921.

39.Emadzade K, Gehrke B, Linder HP, et al. (2011) The biogeographical history of the cosmopolitan genus *Ranunculus* L.(Ranunculaceae) in the temperate to meridional zones. Molecular Phylogenetics and Evolution. 58(1): 4–21.

40.Deng Jia-bin, Bryan T. Drew, Evgeny V. Mavrodiev, Matthew A.Gitzendanner, Pamela S. Soltis, Douglas E. Soltis (2015) Phylogeny, divergence times, and historical biogeography of the angiosperm family Saxifragaceae. Molecular phylogenetics and evolution. 83, 86–98.

41.Zhang JQ, Meng SY, Allen GA, Wen J, Rao GY (2014) Rapid radiation and dispersal out of the Qinghai-Tibetan Plateau of an alpine plant lineage *Rhodiola* (Crassulaceae). Molecular Phylogenetics and Evolution. 77:147–158.

42.Wu Sheng-Dan, Lin Li, Li Hong-Lei, Yu Sheng-Xiang, Zhang Lin-Jing, Wang Wei (2015) Evolution of Asian Interior Arid-Zone Biota: Evidence from the Diversification of Asian *Zygophyllum* (Zygophyllaceae). PloS one, 10(9): 1–17

43.Zhang ML, Huang JF, Sanderson, SC, et al (2015) Molecular biogeography of tribe Thermopsideae (leguminosae): a Madrean-Tethyan disjunction pattern with an African origin of core genistoides. Biomed Research International.

44.Zhang M, Xue J, Zhang Q, et al (2015) Inferring ancestral distribution area and survival vegetation of *Caragana* (Fabaceae) in Tertiary. Plant Systematics and Evolution. 301(7): 1831–1842.

45.Zhang ML, Fritsch PW. (2010) Evolutionary response of *Caragana* (Fabaceae) to Qinghai-Tibetan Plateau uplift and Asian interior aridification. Plant Systematics and Evolution. 288(3-4): 191–199.

46.Zhang ML, Kang Y, Zhong Y, et al (2012) Intense uplift of the Qinghai-Tibetan Plateau triggered rapid diversification of *Phyllolobium* (Leguminosae) in the Late Cenozoic. Plant Ecology and Diversity. 5(4): 491–499.

47.M. Moghaddam, S. Kazempour Osaloo, H. Hosseiny, F. Azimi (2017) Phylogeny and divergence times of the Coluteoid clade with special reference to *Colutea* (Fabaceae) inferred from nrDNA ITS and two cpDNAs, matK and rpl32-trnL(UAG) sequences data. Plant biosystems. 151: 1082–1093.

48.Nasim Azani, Anne Bruneau, Martin F.Wojciechowski, Shahin Zarre (2019) Miocene climate change as a driving force for multiple origins of annual species in *Astragalus* (Fabaceae, Papilionoideae). Molecular Phylogenetics and Evolution.137: 210–221.

49.Zhang ML, Wen ZB, Hao XL, et al (2015) Taxonomy, phylogenetics and biogeography of *Chesneya* (Fabaceae), evidenced from data of three sequences, ITS, trnS-trnG, and rbcL. Biochemical Systematics and Ecology. 63: 80–90.

50.Liu Peiliang (2017) Phylogeography and Biogeography of the genus *Hedysarum* L. (Fabaceae). Northwest Agriculture & Forestry University. Yagling.

51.Zhang Shu-Dong, Jian-Jun Jin, Si-Yun Chen, Mark W. Chase, Douglas E. Soltis, Hong-Tao Li, Jun-Bo Yang, De-Zhu Li, Ting-Shuang Yi (2017) Diversification of Rosaceae since the Late Cretaceous based on plastid phylogenomics. New Phytologist. 214: 1355–1367

52.Gulzar Khan, Fa-Qi Zhang, Qing-Bo Gao, Peng-Cheng Fu, Rui Xing, Jiu-Li Wang, Hai-Rui Liu, Shi-Long Chen (2016) Phylogenetic analyses of Spiraea (Rosaceae) distributed in the Qinghai-Tibetan Plateau and adjacent regions: insights from molecular data. Plant Systematics and Evolution. 302, 11–21.

53.Fu Peng-Cheng, Qing-Bo Gao, Fa-Qi Zhang, Rui Xing, Gulzar Khan, Jiu-Li Wang, Hai-Rui Liu, Shi-Long Chen (2016) Responses of plants to changes in Qinghai–Tibetan Plateau and glaciations: Evidence from phylogeography of a *Sibiraea* (Rosaceae) complex. Biochemical Systematics and Ecology. 65, 72–82.

54.Eugenia Y.Y. Lo, Michael J. Donoghue (2012) Expanded phylogenetic and dating analyses of the apples and their relatives (Pyreae, Rosaceae). Molecular Phylogenetics and Evolution. 63: 230–243.

55.Jin Guihua (2014) Phylogenomics and Biogeography of *Malus* Mill. Kunming Institute of Botany, Chinese Academy of Sciences. Kunming.

56.Ma Yazhen (2013) Phylogeography of the genus *Dasiphora* (Rosaceae) in the Qinghai-Tibetan Plateau. Lanzhou University. Lanzhou.

57.Feng Tao (2017) Systematics and Biogeography of Potentilleae (Rosaceae). Wuhan Botanical Garden, Chinese Academy of Sciences. Wuhan.

58.Dobeš C, Paule J. (2010) A comprehensive chloroplast DNA-based phylogeny of the genus *Potentilla* (Rosaceae): implications for its geographic origin, phylogeography and generic circumscription. Molecular Phylogenetics and Evolution. 56 (1): 156–75.

59.Tao Feng, Michael J. Moore, Min-Hui Yan, Yan-Xia Sun, Hua-Jie Zhang, Ai-Ping Meng, Xiao-Dong Li, Shu-Guang Jian, Jian-Qiang Li, Heng-Chang Wang (2017) Phylogenetic study of the tribe Potentilleae (Rosaceae), with further insight into the disintegration of Sibbaldia. Journal of Systematics and Evolution. 55 (3): 177–191.

60.Zhu Zhang-Ming, Gao Xin-Fen, Fougère-Danezan Marie (2015) Phylogeny of Rosa sections Chinenses and Synstylae (Rosaceae) based on chloroplast and nuclear markers. Molecular Phylogenetics and Evolution. 87: 50–64.

61.Chin S, Shaw J, Haberle R, et al. (2014) Diversification of almonds, peaches, plums and cherries-Molecular systematics and biogeographic history of *Prunus* (Rosaceae). Molecular Phylogenetics and Evolution. 76: 34–48.

62.Jia Dong-Rui, Richard J. Abbott, Teng-Liang Liu, Kang-Shan Mao, Igor V. Bartish, Jian-Quan Liu (2012) Out of the Qinghai-Tibet Plateau: evidence for the origin and dispersal of Eurasian temperate plants from a phylogeographic study of Hippophae¨ rhamnoides (Elaeagnaceae). New Phytologist. 194: 1123–1133.

63.Wu Zeng‐Yuan, Jie Liu, Jim Provan，Hong Wang, Chia‐Jui Chen, Marc W. Cadotte, Ya‐Huang Luo, Bruno S. Amorim, De‐Zhu Li, Richard I. Milne (2018) Testing Darwin's transoceanic dispersal hypothesis for the inland nettle family (Urticaceae). Ecology Letters. 21:1515–1529.

64.Guido W. Grimm, Susanne S. Renner (2013) Harvesting Betulaceae sequences from GenBank to generate a new chronogram for the family. Botanical Journal of the Linnean Society. 172, 465–477.

65.Meseguer AS, Aldasoro JJ, Sanmartín I. (2013) Bayesian inference of phylogeny, morphology and range evolution reveals a complex evolutionary history in St. John’s wort (Hypericum). Molecular Phylogenetics and Evolution. 67(2): 379–403.

66.Nakamura K, Denda T, Kokubugata G, et al. (2015) Phylogeny and biogeography of the Viola iwagawae-tashiroi species complex (Violaceae, section Plagiostigma) endemic to the Ryukyu Archipelago, Japan. Plant systematics and evolution. 301(1): 337–351.

67.Wu J, Nyman T, Wang DC, et al (2015) Phylogeny of *Salix* subgenus Salix s.l. (Salicaceae): delimitation, biogeography, and reticulate evolution. BMC Evolutionary Biology. 15(1): 31.

68.Thomas Marcussen, Andrea S.Meseguer (2017) Species-level phylogeny, fruit evolution and diversification history of *Geranium* (Geraniaceae). Molecular Phylogenetics and Evolution. 110: 134–149.

69.Xie L, Wagner WL, Ree RH, et al. (2009) Molecular phylogeny, divergence time estimates, and historical biogeography of *Circaea* (Onagraceae) in the Northern Hemisphere. Molecular Phylogenetics and Evolution. 53(3): 995–1009.

70.Zhang ML, Temirbayeva K, Sanderson SC, et al (2015) Young dispersal of xerophil Nitraria lineages in intercontinental disjunctions of the Old World. Scientific Reports. 5: 13840.

71.Zhang Yonghong (2007) Study on the breeding system, molecular evolution and modern biogeographical pattern in Stellera chamaejasme. Kunming Institute of Botany, Chinese Academy of Sciences. Kunming

72.Zhang Ticao, Qin Qiao, Polina Yu. Novikova, Qia Wang, Jipei Yue, Yanlong Guan, Shengping Ming, Tianmeng Liu, Ji De, Yixuan Liu, Ihsan A. Al-Shehbaz, Hang Sun, Marc Van Montagu, Jinling Huang, Yves Van de Peer, La Qiong (2019) Genome of Crucihimalaya himalaica, a close relative of Arabidopsis, shows ecological adaptation to high altitude. Proceedings of the National Academy of Sciences. 116 (14): 7137–7146.

73.Chen S, Wu G, Chen S, et al. (2010) Molecular phylogeny and biogeography of the narrow endemic Coelonema and affinitive *Draba* (Brassicaceae) based on two DNA regions. Biochemical Systematics and Ecology. 38(4): 796–805.

74.Friesen N, German DA, Hurka H, et al (2016) Dated phylogenies and historical biogeography of *Dontostemon* and *Clausia* (Brassicaceae) mirror the palaeogeographical history of the Eurasian steppe. Journal of Biogeography. 43(4): 738–749.

75.Yue JP, Sun H, Baum DA, et al (2009) Molecular phylogeny of *Solms-laubachia* (Brassicaceae) sl, based on multiple nuclear and plastid DNA sequences, and its biogeographic implications. Journal of Systematics and Evolution. 47(5): 402–415.

76.Mingli Zhang, Xiaoli Hao, Stewart C. Sanderson, Byalt V. Vyacheslav, Alexander P. Sukhorukov, Xia Zhang (2014) Spatiotemporal evolution of *Reaumuria* (Tamaricaceae) in Central Asia: insights from molecular biogeography. Phytotaxa.167 (1): 089–103.

77.Zhang ML, Meng HH, Zhang HX, et al (2014) Himalayan origin and evolution of *Myricaria* (Tamaricaeae) in the neogene. PloS one. 9(6): e97582.

78.Lledó MD, Crespo MB, Fay MF, et al (2005) Molecular phylogenetics of *Limonium* and related genera (Plumbaginaceae): biogeographical and systematic implications. American Journal of Botany. 92(7): 1189–1198.

79.Fan DM, Chen JH, Meng, Y, et al (2013) Molecular phylogeny of *Koenigia* L.(Polygonaceae: Persicarieae): implications for classification, character evolution and biogeography. Molecular Phylogenetics and Evolution. 69(3): 1093–1100.

80.M.Talavera, F.Balao, R. Casimiro-Soriguer, M. Á. Ortiz, A.Terrab, M. Arista, P. L. Ortiz, T. F. Stuessy, S. Talavera (2011) Molecular phylogeny and systematics of the highly polymorphic *Rumex* *bucephalophorus* complex (Polygonaceae). Molecular Phylogenetics and Evolution.61 (3): 659–670.

81.Sun Y, Wang A, Wan D, et al (2012) Rapid radiation of *Rheum* (Polygonaceae) and parallel evolution of morphological traits. Molecular Phylogenetics and Evolution. 63(1): 150–158.

82.Daniel B Sloan, Bengt Oxelman, Anja Rautenberg, Douglas R Taylor (2009) Phylogenetic analysis of mitochondrial substitution rate variation in the angiosperm tribe Sileneae. BMC Evolutionary Biology. 9: 260.

83.Anna Petri, Bernard E. Pfeil, Bengt Oxelman (2013) Introgressive Hybridization between Anciently Diverged Lineages of *Silene* (Caryophyllaceae). PloS One. 8(7): e67729.

84.Valente LM, Savolainen V, Vargas P. (2010) Unparalleled rates of species diversification in Europe. Proceedings of the Royal Society B: Biological Sciences. 277(1687): 1489–1496.

85.Guo YL, Pais A, Weakley AS, et al (2013) Molecular phylogenetic analysis suggests paraphyly and early diversification of *Philadelphus* (Hydrangeaceae) in western North America: New insights into affinity with Carpenteria. Journal of Systematics and Evolution. 51(5): 545–563.

86. Janssens SB, Knox EB, Huysmans S, et al (2009) Rapid radiation of *Impatiens* (Balsaminaceae) during Pliocene and Pleistocene: result of a global climate change. Molecular Phylogenetics and Evolution. 52(3): 806–824.

87.Florian C. Boucher, Niklaus E. Zimmermann, Elena Conti (2016) Allopatric speciation with little niche divergence is common among alpine Primulaceae. Journal of Biogeography. 43: 591–602.

88.Guangpeng Ren, Elena Conti, Nicolas Salamin (2015) Phylogeny and biogeography of *Primula* sect. Armerina: implications for plant evolution under climate change and the uplift of the Qinghai-Tibet Plateau. BMC Evolutionary Biology. 15:161–176.

89.Guangpeng Ren, Rubén G. Mateo, Antoine Guisan, Elena Conti, Nicolas Salamin (2018) Species divergence and maintenance of species cohesion of three closely related Primula species in the Qinghai-Tibet Plateau. Journal of Biogeography. 45: 2495–2507.

90.Wang YuJin, Li Xiaojuan, Hao Gang, Liu Jianquan (2004) Molecular phylogeny and biogeography of *Androsace* (Primulaceae) and the convergent evolution of cushion morphology. Acta Phytotaxonomica Sinica. 42 (6) : 481–499.

91.Gai-Ni Wang, Xin-Yu He, Georg Miehe, Kang-Shan Mao (2014) Phylogeography of the Qinghai–Tibet Plateau endemic alpine herb *Pomatosace filicula* (Primulaceae). Journal of Systematics and Evolution.52 (3): 289–302.

92.Zhang Lin (2013) Mocular Phylogeny of Ericoideae and Microsatellite Markers Development for Diplarche multiflora. Kunming Institute of Botany, Chinese Academy of Sciences. Kunming.

93.Von Hagen KB, Kadereit JW, Williams CF. (2003) The diversification of *Halenia* (Gentianaceae): ecological opportunity versus key innovation. Evolution. 57(11): 2507–2518.

94.Favre A, Michalak I, Chen CH, et al. (2016) Out-of-Tibet: the spatio-temporal evolution of *Gentiana* (Gentianaceae). Journal of Biogeography. 43: 1967–1978.

95.Wen‐Tao Yu, Frédéric M. B. Jacques, Shao‐Tian Chen, Zhe‐Kun Zhou (2012) Nutlet micro-morphology of the genus *Microula* (Boraginaceae) from the Qinghai-Tibetan Plateau, and its systematic implications. Nordic Journal of Botany. 30(5):596–612.

96.Thomas C. Mitchell , Bethany R. M. Williams , John R. I. Wood (2016) How the temperate world was colonised by bindweeds: biogeography of the Convolvuleae (Convolvulaceae). BMC Evolutionary Biology. 16: 16.

97.Tu Tieyao (2008) Phylogeny and Biogeography of the tribes Nolaneae,Hyoscyameae and Mandragoreae of Solanaceae. Kunming Institute of Botany, Chinese Academy of Sciences. Kunming.

98.Tu, TY, Volis, S, Dillon, MO, et al. (2010) Dispersals of Hyoscyameae and Mandragoreae (Solanaceae) from the New World to Eurasia in the early Miocene and their biogeographic diversification within Eurasia. Molecular Phylogenetics and Evolution. 57(3): 1226–1237.

99.Jin-Ming Chen, Zhi-Yuan Du, Shan-Shan Sun, Robert Wahiti Gituru, Qing-Feng Wang (2013) Chloroplast DNA Phylogeography Reveals Repeated Range Expansion in a Widespread Aquatic Herb *Hippuris vulgaris* in the Qinghai-Tibetan Plateau and Adjacent Areas. Plos One. 8(4): e60948.

100.Li GD, Kim C, Zha HG, et al (2014) Molecular phylogeny and biogeography of the arctic-alpine genus *Lagotis* (Plantaginaceae). Taxon. 63(1): 103–115.

101.Luo Dong (2015) Phylogeographic studies of four perennial herbs endemic to the subnival zone of the QTP. Kunming Institute of Botany, Chinese Academy of Sciences.

102.Shaotian Chen, Yaowu Xing, Tao Su, Zhekun Zhou, Emeritus David L Dilcher, Douglas E Soltis (2012) Phylogeographic analysis reveals significant spatial genetic structure of *Incarvillea sinensis* as a product of mountain building. BMC Plant Biology.12: 58.

103.Hong-Hu Meng, Ming-Li Zhang (2013) Diversification of plant species in arid Northwest China: Species-level phylogeographical history of *Lagochilus* Bunge ex Bentham (Lamiaceae). Molecular Phylogenetics and Evolution. 68: 398–409

104.Yu XQ, Maki M, Drew BT, et al. (2014) Phylogeny and historical biogeography of *Isodon* (Lamiaceae): Rapid radiation in south-west China and Miocene overland dispersal into Africa. Molecular Phylogenetics and Evolution. 77: 183–194.

105.Tian Zunzhe (2017) Phylogeography of *Lancea tibetica* on the Qinghai-Tibetan Plateau. University of Chinese Academy of Sciences. Beijing.

106.Gussarova G, Popp M, Vitek E, et al. (2008) Molecular phylogeny and biogeography of the bipolar *Euphrasia* (Orobanchaceae): recent radiations in an old genus. Molecular Phylogenetics and Evolution. 48 (2): 444–460.

107.Andrea D. Wolfe, Christopher P. Pandle, Liang Liu, Kim E. Steiner (2005) Phylogeny and Biogeography of Orobanchaceae. Folia Geobotanica. 40: 115–134.

108.Wang HJ., Li WT., Liu YN., Yang FS., Wang XQ. (2015) Range-wide multilocus phylogenetic analyses of *Pedicularis* sect. Cyathophora (Orobanchaceae): implications for species delimitation and speciation. Taxon. 64, 959–974.

109.Zhou Z, Hong D, Niu Y, et al (2013) Phylogenetic and biogeographic analyses of the Sino-Himalayan endemic genus *Cyananthus* (Campanulaceae) and implications for the evolution of its sexual system. Molecular Phylogenetics and Evolution. 68: 482–497.

110.Liu JQ, Gao TG, Chen ZD, et al (2002) Molecular phylogeny and biogeography of the Qinghai-Tibet Plateau endemic *Nannoglottis* (Asteraceae). Molecular Phylogenetics and Evolution. 23(3): 307–325.

111.Nie ZL, Funk VA, Meng Y, et al (2016) Recent assembly of the global herbaceous flora: evidence from the paper daisies (Asteraceae: Gnaphalieae). New Phytologist. 209: 1795–1806.

112.Sanz M, Schneeweiss GM, Vilatersana R, et al (2011) Temporal origins and diversification of Artemisia and allies (Anthemideae, Asteraceae). Collectanea Botanica. 30: 7–15.

113.Jian-Quan Liu, Yu-Jing Wang, Ai-Lan Wang, Ohba Hideaki, Richard J. Abbott (2006) Radiation and diversiWcation within the Ligularia–Cremanthodium–Parasenecio complex (Asteraceae) triggered by uplift of the Qinghai-Tibetan Plateau. Molecular Phylogenetics and Evolution. 38: 31–65.

114.Wang, YJ, Susanna, A, Von Raab-Straube, E, et al (2009) Island-like radiation of Saussurea (Asteraceae: Cardueae) triggered by uplifts of the Qinghai–Tibetan Plateau. Biological Journal of the Linnean Society. 97(4): 893–903.

115.Wang YJ, Liu JQ, Miehe G. (2007) Phylogenetic origins of the Himalayan endemic Dolomiaea, Diplazoptilon and Xanthopappus (Asteraceae: Cardueae) based on three DNA regions. Annals of Botany. 99 (2): 311–322.

116.Bell, CD, Mavrodiev, EV, Soltis, PS, et al. (2012) Rapid diversification of Tragopogon and ecological associates in Eurasia. Journal of Evolutionary Biology. 25(12): 2470–2480.

117.Zhang JW, Nie ZL, Wen J, Sun H (2011) Molecular phylogeny and biogeography of three closely related genera, Soroseris, Stebbinsia, and Syncalathium (Asteraceae, Cichorieae), endemic to the Tibetan Plateau, SW China. Taxon. 2011; 60(1): 15–26.

118.Zhang JW, Boufford DE, Sun H (2011) Parasyncalathium JW Zhang, Boufford & H. Sun (Asteraceae, Cichorieae): A new genus endemic to the Himalaya-Hengduan Mountains. Taxon. 60(6): 1678–1684.

119.Smith SA, Donoghue MJ (2010) Combining historical biogeography with niche modeling in the Caprifolium clade of Lonicera (Caprifoliaceae, Dipsacales). Systematic Botany. 59(3): 322–341.

120.Rong Li, Jun Wen (2016) Phylogeny and diversification of Chinese Araliaceae based on nuclear and plastid DNA sequence data. Journal of Systematics and Evolution. 54 (4): 453–467.

121.Filip Vandelook, Steven B. Janssens, Robin J. Probert (2012) Relative embryo length as an adaptation to habitat and lifecycle in Apiaceae. New Phytologist. 195: 479–487.

122.Łukasz Banasiak, Marcin Piwczy nski, Tomasz Uli nski, Stephen R.Downie, Mark F. Watson, Bandana Shakya, Krzysztof Spalik (2013) Dispersal patterns in space and time: a case study of Apiaceae subfamily Apioideae. Journal of Biogeography. 40: 1324–1335.

123.He Yanling (2018) Speciation and Evolutionary history of the genus Notopterygium. Northwest University. Xi'an

124.Liao CY, Downie SR, Yu Y, et al. (2012) Historical biogeography of the Angelica group (Apiaceae tribe Selineae) inferred from analyses of nrDNA and cpDNA sequences. Journal of Systematics and Evolution. 50(3): 206–217.
