# Supplementary material for: Current biogeographical roles of the Kunlun Mountains
Source: Ecol Evol. 2022 Jan 15;12(1):e8493. doi: 10.1002/ece3.8493 (PMC8809438; doi:10.1002/ece3.8493)
Supplement: Supplementary file 5 — Appendix S4 [file ECE3-12-e8493-s002.docx]

Appendix Table A1-2.

**Appendix Table A 1** The county area and abbreviation of the Kunlun Mountains.

| **East** **Kunlun Mountains** | | | **North slope of Middle Kunlun Mountains** | | |
| --- | --- | --- | --- | --- | --- |
| Counties | Area (km^2^) | Abbreviation | Counties | Area (km^2^) | Abbreviation |
| Banma | 6,500 | BM | Dulan | 21,000 | DL |
| Jiuzhi | 8,700 | JZ | Geermu | 22,000 | GEM |
| Dari | 16,000 | DR | Ruoqiang | 20,000 | RQ |
| Gande | 7,000 | GD | Qiemo | 23,000 | QM |
| Chenduo | 17,000 | CD | Minfeng | 18,000 | MF |
| Maduo | 23,000 | MD | Yutian | 17,000 | YT |
| Maqin | 14,000 | MQ | Cele | 20,000 | CL |
| Xinghai | 12,000 | XH | Hetian | 20,000 | HT |
|  | 104,200 |  |  | 16,100 |  |
| **South slope of Middle Kunlun Mountains** | | | **West Kunlun Mountains** | | |
| Counties | Area (km^2^) | Abbreviation | Counties | Area (km^2^) | Abbreviation |
| Qumalai | 23,000 | QML | Pishan | 20,000 | PS |
| Zhiduo | 23,000 | ZD | Yecheng | 22,000 | YC |
| Bange | 20,000 | BG | Shache | 16,000 | SC |
| Nima | 18,000 | NM | Taxian | 23,000 | TX |
| Gaize | 16,000 | GZ | Aketao | 23,000 | AKT |
| Ritu | 20,000 | RT | Wuqia | 22,000 | WQ |
|  | 120,000 |  |  | 126,000 |  |

The mountain range covers a total area of more than 500,000 km^2^. To accurately reveal the current plant diversity, the study region was divided into 28 county-level geographical units according to the county area and vegetation type (Supplementary Table 1). The area of 25 units are not significantly different on the Kunlun Mountains (Supplementary Table 1). In the southeastern section of the Kunlun Mountains, the area of 3 adjacent units (Banma, Jiuzhi, Gande) are less than 10,000 km^2^ (Supplementary Table 1). However, the vegetation types of the 3 adjacent units are obviously different. The coniferous forests are concentrated in Banma, the Jiuzhi is dominated by alpine scrub, the vegetation type of Gande is alpine meadow. The 3 adjacent units were significant for further revealing further community phylogenetic structure in the eastern section of the Kunlun Mountains. Therefore, the 3 adjacent units is not merged, the division of the study region is suitable.

To further confirm conservation value of the east Kunlun Mountains, we have merged adjacent units which are less than 10,000 km^2^. Banma and Jiuzhi were merged, named Banjiu. Gande and Maqin were merged, named Ganma. After the merger, the area of 6 units are not significantly different on the east Kunlun Mountains (Supplementary Table 2). In addition, priority area of conservation was more definite according to the genera richness.

**Appendix Table A 2** Number of species and genera on the east Kunlun Mountains.

| **Counties** | **Species** | **Genera** | **MDT** | **SES-MDT** | **P-value** | **Area (km^2^)** |
| --- | --- | --- | --- | --- | --- | --- |
| Banjiu | 751 | 250 | 21.79 |  |  | 15,200 |
| Dari | 288 | 119 | 19.61 | -0.06 | 0.465 | 16,000 |
| Ganma | 758 | 237 | 20.93 |  |  | 21,000 |
| Chenduo | 490 | 179 | 19.74 | 0.20 | 0.414 | 17,000 |
| Maduo | 471 | 140 | 19.44 | -0.42 | 0.356 | 23,000 |
| Xinghai | 731 | 236 | 18.87 | 0.46 | 0.322 | 12,000 |
