# Supplementary material for: Current biogeographical roles of the Kunlun Mountains
Source: Ecol Evol. 2022 Jan 15;12(1):e8493. doi: 10.1002/ece3.8493 (PMC8809438; doi:10.1002/ece3.8493)
Supplement: Supplementary file 6 — Supplementary Material [file ECE3-12-e8493-s004.docx]

**Appendix figures and tables**

**Figures**

**Appendix S1 Fig. A1** The phylogeny of seed plants in the Kunlun Mountains.

**Tables**

**Appendix Table A 1** The county area and abbreviation of the Kunlun Mountains.

**Appendix Table A 2** Number of species and genera on the east Kunlun Mountains.

**Excel Tables**

**Appendix 1** Seed plants of Mt. Kunlun_Data.

**Appendix 2** Seed plants of counties in Mt. Kunlun_Data.
